# Sex-Specific Role for Dopamine Receptor D2 in Dorsal Raphe Serotonergic Neuron Modulation of Defensive Acoustic Startle and Dominance Behavior

**DOI:** 10.1523/ENEURO.0202-20.2020

**Published:** 2020-12-23

**Authors:** Krissy A. Lyon, Benjamin D. Rood, Lorna Wu, Rebecca A. Senft, Lisa V. Goodrich, Susan M. Dymecki

**Affiliations:** 1 Department of Genetics, Harvard Medical School, Boston, MA 02115; 2 Department of Neurobiology, Harvard Medical School, Boston, MA 02115

**Keywords:** acoustic startle, dominance, dopamine receptor, raphe, serotonin, sex differences

## Abstract

Brain networks underlying states of social and sensory alertness are normally adaptive, influenced by serotonin and dopamine (DA), and abnormal in neuropsychiatric disorders, often with sex-specific manifestations. Underlying circuits, cells, and molecules are just beginning to be delineated. Implicated is a subtype of serotonergic neuron denoted *Drd2-Pet1*, distinguished by expression of the type-2 DA receptor (*Drd2*) gene, inhibited cell-autonomously by DRD2 agonism in slice, and, when constitutively silenced in male mice, affects levels of defensive and exploratory behaviors (Niederkofler et al., 2016). Unknown has been whether DRD2 signaling in these *Pet1* neurons contributes to their capacity for shaping defensive behaviors. To address this, we generated mice in which *Drd2* gene sequences were deleted selectively in *Pet1* neurons. We found that *Drd2^Pet1-CKO^* males, but not females, demonstrated increased winning against sex-matched controls in a social dominance assay. *Drd2^Pet1-CKO^* females, but not males, exhibited blunting of the acoustic startle response, a protective, defensive reflex. Indistinguishable from controls were auditory brainstem responses (ABRs), locomotion, cognition, and anxiety-like and depression-like behaviors. Analyzing wild-type *Drd2-Pet1* neurons, we found sex-specific differences in the proportional distribution of axonal collaterals, in action potential (AP) duration, and in transcript levels of *Gad2,* important for GABA synthesis. *Drd2^Pet1-CKO^* cells displayed sex-specific differences in the percentage of cells harboring *Gad2* transcripts. Our results suggest that DRD2 function in *Drd2-Pet1* neurons is required for normal defensive/protective behaviors in a sex-specific manner, which may be influenced by the identified sex-specific molecular and cellular features. Related behaviors in humans too show sex differences, suggesting translational relevance.

## Visual Abstract

**Figure:**
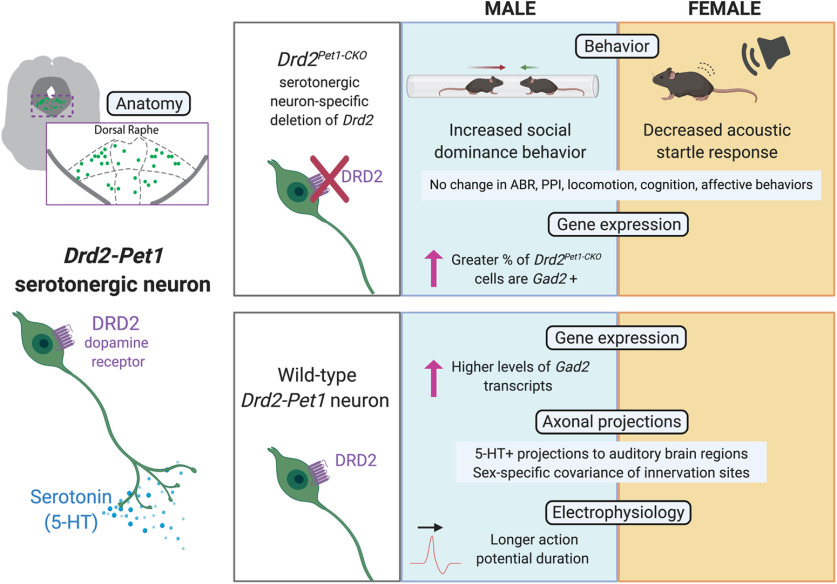

## Significance Statement

A subtype of dorsal raphe (DR) serotonergic neuron, denoted *Drd2-Pet1*, is poised for regulation by dopamine (DA) via type-2 DA receptor (DRD2) expression. Functional removal of DRD2 in these cells through a conditional knockout (CKO) mouse strategy resulted in sex-specific behavioral abnormalities: *Drd2^Pet1^*^-CKO^ females exhibited reduced acoustic startle while males showed increased social dominance. *Drd2-Pet1* neurons were similar in number and distribution in males versus females but exhibited sex-specific differences in neurotransmission-related mRNAs, action potential (AP) duration, and relative distribution of collaterals. Abnormalities in sensory processing and social behaviors akin to those reported here manifest in autism, schizophrenia, and posttraumatic stress disorder, in sex-specific ways. Our findings, thus, may point to novel circuits and modulatory pathways relevant to human neuropsychiatric conditions.

## Introduction

The serotonergic and dopaminergic neurotransmitter systems are known for their influence on and maladaptation in neuropsychiatric disorders, including posttraumatic stress disorder, autism spectrum disorder, and schizophrenia. Clinical and animal studies implicate serotonin (5-hydroxytryptamine; 5-HT) and dopamine (DA) in modulation of endophenotypes common to neuropsychiatric disorders, such as altered social interaction and sensory processing (Geyer and Braff, 1987; Meincke et al., 2004; Takahashi and Kamio, 2018). Transcriptome data coupled with structure-function maps in mice show that the serotonergic and dopaminergic neuronal systems are themselves heterogeneous, comprised of functionally specialized neuronal subtypes, manifesting distinct mRNA profiles, efferent projections, electrophysiological properties, and functions (Jensen et al., 2008; Kim et al., 2009; Crawford et al., 2013; Lammel et al., 2014; Spaethling et al., 2014; Okaty et al., 2015; Deneris and Gaspar, 2018; Poulin et al., 2018, 2020; Huang et al., 2019; Ren et al., 2019; Okaty et al., 2020). An important subtype of serotonergic neuron as relates to social and defensive behaviors is denoted *Drd2-Pet1* (Niederkofler et al., 2016), identified by expression of the type-2 DA receptor (*Drd2*) gene and the serotonergic transcription factor gene *Pet1 (*aka *Fev)*. DRD2 agonism in slice preparation drove outward (inhibitory) currents cell-autonomously in *Drd2-Pet1* neurons, suppressing their excitability; and when these cells were constitutively silenced in male mice, i.e., exocytic neurotransmitter release was cell autonomously blocked, defensive, aggressive, and exploratory behaviors increased (Niederkofler et al., 2016). Here, we query whether *Drd2* expression in *Drd2-Pet1* cells contributes to the modulation of defensive, exploratory behaviors.

While *Drd2* is expressed in many cell types throughout the midbrain and basal forebrain, expression in serotonergic neurons is restricted to a small subset of cells resident in the dorsal raphe (DR) nucleus. In these serotonergic neurons, *Drd2* expression initiates around adolescence and continues through adulthood, at which point, *Drd2* transcripts are the major DA receptor mRNA detected (Niederkofler et al., 2016). Thus, *Drd2-Pet1* neurons come under DRD2 and presumably DA regulation during the developmental transition to sexual maturity. *Drd2-Pet1* neurons project to brain regions involved in sensory processing, defensive, and mating behaviors including auditory brainstem regions and the sexually dimorphic medial preoptic area (mPOA; Niederkofler et al., 2016). These findings led us to hypothesize that DRD2 signaling in *Drd2-Pet1* neurons contributes to social and sensory alertness and defensive behavior in a sex-specific manner.

Indeed, serotonergic and dopaminergic perturbations affect social and defensive behaviors differently in male versus female rodents. Decreases in serotonergic tone associate with increased levels of aggression in males (Brown et al., 1982; Hendricks et al., 2003; Yu et al., 2014; Niederkofler et al., 2016). By contrast, lesions of the serotonergic DR in female rats decreased maternal aggression (Holschbach et al., 2018), while DR serotonergic neuron activity in female, but not male, hamsters associates with social dominance (Terranova et al., 2016). The acoustic startle reflex (ASR), an evolutionarily-conserved, defensive reflex to loud, potentially threatening stimuli (Davis et al., 1982), also shows sex-specific differences within the context of altered 5-HT levels. Reduction in 5-HT levels enhanced ASR in female but not male rats (Pettersson et al., 2016). With respect to DA, deletion of the DA re-uptake transporter gene (*Dat*) altered ASR only in male mice (Ralph et al., 2001). Genetic removal of the soluble form of catechol-O-methyltransferase (COMT), important for degradation of DA, enhanced the ASR and dominance behaviors in both sexes, but ASR especially in males (Tammimäki et al., 2010). Thus, serotonergic and dopaminergic neuronal systems influence social behaviors and sensory processing in sex-specific ways.

Here, we queried whether *Drd2* conditional deletion in serotonergic neurons would alter aggression and social dominance behavior in males. Further, we sought to examine the role of *Drd2* expression in serotonergic neurons in females with the hypothesis that other sensory or defensive behaviors would be affected, given typical lack of aggression in female mice (Lonstein and Gammie, 2002). We undertook a phenotypic analysis of mice in which we engineered *Drd2* gene deletion selectively in *ePet1-cre*-expressing serotonergic neurons (*Drd2^Pet1^*^-CKO^ mice). Here, we report that *Drd2^Pet1^*^-CKO^ males exhibited increased social dominance whereas females displayed a robust decrease in ASR. We also investigated sex differences in *Drd2-Pet1* neurons at the molecular, cellular, and circuit levels, identifying differences in candidate mRNA levels, electrophysiological properties, and relative distribution densities of axonal collaterals.

## Materials and Methods

### Ethical approval

All experimental protocols were approved by Harvard University Institutional Animal Care and Use Committees (IACUC) and were in accordance with the animal care guidelines of the National Institutes of Health.

### Experimental animals

Mice were housed in a temperature-controlled environment on a 12/12 h light/dark cycle with *ad libitum* access to standard mouse chow and water. All experimental animals were virgins. For conditional knockout of *Drd2,* double transgenic mice of the genotype *ePet-Cre;Drd2^loxP/loxP^* (referred to as *Drd2^Pet1^*^-CKO^) were generated by crossing BAC transgenic *ePet-Cre* (Scott et al., 2005; Jax #012712) males to homozygous *Drd2^loxP/loxP^* (Bello et al., 2011; Jax #020631) females. From these crosses, *ePet-Cre;Drd2^loxP/wild-type^* males were then bred to homozygous *Drd2^loxP/loxP^* females for *ePet-Cre;Drd2^loxP/loxP^* male and female offspring used for experiments. Experimental controls were littermates with the *Drd2^loxP/loxP^* genotype thus negative for Cre but of comparable genetic background (C57BL/6J, Jax #000664). For *Drd2-Pet1* neuron cell counts, triple transgenic *Drd2-Cre;Pet1-Flpe;RC-FrePe* (Gong et al., 2007; Jensen et al., 2008; Brust et al., 2014; *RC-FrePe* Jax #029486) were generated by crossing *Drd2-Cre* females to *Pet1-Flpe;RC-FrePe* double transgenic males. Likewise for axonal projection mapping, *Drd2-Cre; Pet1-Flpe; RC-FPSit* (*RC-FPSit* Jax #030206) triple transgenic mice were generated by crossing *Drd2-Cre* females to *Pet1-Flpe;RC-FPSit* double transgenic males. For both *RC-FrePe* and *RC-FPSit* crosses, all animals of each sex were from separate litters, though males and females from the same litter were used when possible. Genotypes were determined as previously described (Brust et al., 2014). Number of animals used for each assay is listed under the description for each assay.

### Immunohistochemistry

Mice were briefly anesthetized with isoflurane and immediately perfused intracardially with PBS followed by 4% paraformaldehyde (PFA) in PBS. Brains were extracted, postfixed in 4% PFA overnight at 4°C, cryoprotected in 30% sucrose/PBS for 48 h, and embedded in OCT compound (Tissue-Tek). Coronal sections were cryosectioned as 30-μm free-floating sections then rinsed three times with PBS for 10 min, blocked in 5% normal donkey serum (NDS; Jackson ImmunoResearch) and permeabilized with 0.1% Triton X-100 in PBS for 1 h at room temperature. Sections were incubated for 24–48 h in primary antibodies in the same blocking buffer at 4°C. Primary antibodies used were goat polyclonal anti-5-HT (1:1000, catalog #ab66047; Abcam), chicken polyclonal anti-GFP (1:2000, RRID: AB_2307313; AVES), rabbit polyclonal anti-DsRed (1:1000; catalog #632496; Clontech), and rabbit anti-GABA (1:500, catalog #A2052; Sigma). Following primary antibody incubation, sections were rinsed three times with PBS for 10 min and incubated in secondary antibody (Alexa Fluor 488 donkey anti-chicken IgY, 703-545-155, Jackson ImmunoResearch; Alexa Fluor 546 donkey anti-rabbit IgG, A10040, Invitrogen; Alexa Fluor 647 donkey anti-goat IgG, A-21447, Invitrogen) for 1 h at room temperature, rinsed three times with PBS for 10 min, then mounted using ProLong Gold Antifade Mountant (P36930, LifeTechnologies). For *Drd2-Pet1* neuron cell counts, GFP+ cells were counted in every sixth section. The resulting number was multiplied by 6 to obtain the number of *Drd2-Pet1* cells per animal.

### Dual immunohistochemistry and fluorescent *in situ* hybridization (FISH)

For dual *in situ* hybridization with immunostaining for GFP+ *Drd2-Pet1* neuron cell bodies, PFA-perfused brain tissue from adult *Drd2-Cre;Pet1-Flpe;RC-FrePe* mice was collected as described above but cryosectioned at 20 μm onto slides (Superfrost Plus, catalog #48311-703, VWR), slides were warmed on a slide warmer set to 45°C for 30 min, and processed with RNAscope Multiplex Fluorescent Assay kit (Advanced Cell Diagnostics) following manufacturer’s protocol with the exception that at the end of the protocol, tissue was stained for anti-GFP, as described above, similar to Shrestha et al. (2018). The following probes were used for the dual protocol: *Dmd* (catalog #561551-C3), *Drd2-E2* (catalog #486571-C2), *Gad2* (catalog #439371-C2), and *Serpini1* (catalog #501441). Cell nuclei were visualized with 4’,6-diamidino-2-phenylindole (DAPI).

### FISH

For FISH validation of *Drd2* conditional knockout and *Gad2* expression analysis, adult *Drd2^Pet1-CKO^* or control brain tissue was fresh frozen in OCT (TissueTek) and cryosectioned at 16 μm onto slides (Superfrost Plus, catalog #48311-703, VWR) and then processed with RNAscope Multiplex Fluorescent Assay kit (Advanced Cell Diagonstics) following manufacturer’s protocol for fresh frozen tissue. The following probes were used: *Drd2-E2* (catalog #486571-C2), *Drd2-*O4 (Exon7/8; catalog #534241), *Fev (Pet1)* (catalog #413241-C3), *Gad2* (catalog #439371-C2), *Tph2* (catalog #318691), and *cre* (catalog #312281). Cell nuclei were visualized with DAPI.

### Image collection

All images were acquired on a Nikon Ti inverted spinning disk confocal microscope with 488-, 561-, 647-nm laser lines and Andor Zyla 4.2 Plus sCMOS monochrome camera. Images were acquired with Nikon Elements Acquisition software AR 5.02. For RNA quantification and *Drd2^Pet1-CKO^* validation experiments, four images were taken of brain slices containing the DR: the first directly ventral to the aqueduct then one field of view below and to the left and right to capture each lateral wing.

### FISH quantification

Quantification was conducted blind to sex and genotype. For *Drd2^Pet1^*^-CKO^ validation, all *Pet1+* (serotonergic) neurons within each image were identified, then the viewer outlined the DAPI-stained nuclei of each *Pet1+* neuron and scored the presence of *Drd2* puncta as “positive” (having puncta) or “negative” (no puncta).The total number of *Drd2+ Pet1+* neurons was then divided by the total number of *Pet1+* neurons to yield the “% *Drd2+Pet1*+ neurons.”

For quantification of *Dmd*, *Drd2*, *Gad2*, and *Serpini1* manual counting of each mRNA punctum per cell was conducted by a trained viewer. All cells counted fit the criteria of GFP+ with a DAPI+ nucleus. The viewer outlined the GFP+ cell body in FIJI (https://Fiji.sc/; Schindelin et al., 2012) while only viewing that channel and then counted the number of distinct RNA puncta within that cell outline. Brain sections sampled were from five males and five female animals.

For quantification of *Drd2-*Exon7/8 and *Gad2* puncta in *Drd2^Pet1^*^-CKO^ tissue, DR sections corresponded to interaural −0.80 to 1.04 mm and bregma −4.60 to – 4.84 mm based on DAPI staining and anatomic landmarks (Franklin and Paxinos, 2008), where *Drd2-Pet1* neurons are most enriched. A series of custom FIJI scripts and a CellProfiler (McQuin et al., 2018) pipeline were used to process and analyze confocal images of RNAscope FISH signal in a semi-automatic manner. Analysis was performed in 2D on maximum intensity projections of 6-µm-thick *z*-stacks. First, a (step 1) preprocessing FIJI script separated channels and preprocessed them for (step 2) CellProfiler to use as input to segment nuclei. The DAPI-stained channel was preprocessed by a Gaussian blur with a diameter of 18 before segmenting with the IdentifyPrimaryObjects module with a diameter range 30–100 pixels using a minimum cross entropy global thresholding strategy. Objects outside of the diameter range or those on the edges were excluded. A threshold smoothing scale of 1.3488 was used and the image was automatically declumped based on intensity values. Finally, holes were filled in the resulting label map image, which was exported for use in FIJI (step 3). In FIJI, the user manually excluded misidentified objects or added additional nuclei that were missed by the automatic detection pipeline. A highly similar script was recently published (Okaty et al., 2020), though this current script performs additional difference of Gaussian (Marr and Hildreth, 1980) based filtering for each FISH channel. For each FISH probe, after background subtraction with a rolling ball radius of 50 pixels, the image was duplicated and a Gaussian blur was performed at two different σ levels, one which obscured small background pixels but preserved mRNA puncta, and a more extreme blur that only retained larger diffuse background puncta. The difference of these two images was then calculated and puncta localized using the Find Maxima function. To find appropriate settings for each FISH channel, we compared the performance of several sets of parameters to automatically detect puncta versus a hand count of puncta. We were able to achieve excellent concordance between the hand count and automatic puncta detection. Table 1 summarizes our settings and performance in a linear regression against the hand count for each FISH probe (statistics calculated in GraphPad Prism v8.4.3 and Microsoft Excel v2002).

**Table 1:** Settings for *Gad2* quantification in *Drd2^Pet1^*^-CKO^ tissue

Probe	S1	S2	Prominence	*R*^2^	RMSE	MAE
E2	0.25	1	175	0.8696	0.5957	0.2458
E7/8	0.5	1	100	0.9421	0.8054	0.35
Cre	0.5	1	100	0.9679	3.829	2.2244
Fev	0.25	2	75	0.9555	4.414	3.7047
Gad2	0.25	16	150	0.8568	2.804	1.7973

Summary of settings and performance in a linear regression for semi-automated protocol versus hand counts for each FISH probe.

### Behavioral assays

All assays, except the resident-intruder assay, were conducted in an initial cohort of 15 control (eight males, seven females) and 11 *Drd2^Pet1-CKO^* (six males, five females) mice. All behavioral assays were conducted at postnatal day (P)90 or later. The run order for the initial cohort was open field, elevated plus maze, tail suspension test, forced swim test, social interaction, acoustic startle response, prepulse inhibition of acoustic startle, water T-maze, contextual fear conditioning, tube test of social dominance and rotarod. An additional cohort of 16 controls (seven males, nine females) *Drd2^Pet1-CKO^*(six males, 10 females) was run for acoustic startle response. Resident-intruder assay of aggression was conducted in three separate cohorts of mice totaling 24 control and 26 *Drd2^Pet1-CKO^* males. The tube test of social dominance was run in the initial cohort and in the second (eight control and *Drd2^Pet1-CKO^* males) and third (11 control and *Drd2^Pet1-CKO^* males) aggression cohorts for a total of 24 control and *Drd2^Pet1-CKO^* males and a separate cohort of 16 control and 18 *Drd2^Pet1-CKO^* females. The rotarod assay was also repeated in a separate cohort of males (seven controls, six *Drd2^Pet1-CKO^*). Experiments were conducted between zeitgeber time (ZT)6 and ZT10, with interspersion of control and experimental animals, and assays were run and analyzed by a trained experimenter blinded to genotype. The open field test, elevated plus maze, tail suspension test, forced swim test, social interaction, prepulse inhibition of acoustic startle, water T-maze, and contextual fear conditioning were performed as previously described (Niederkofler et al., 2016). All other behavioral assays are described in detail below.

#### Rotarod

The rotarod apparatus (Stoelting; Ugo Basile Apparatus) contains a rotating rod set to an accelerating speed. Mice are placed onto the rod and rotation of the rod begins. When a mouse loses its balance and falls, the apparatus automatically stops and measures the latency and rotating speed at which the mouse fell. Training consisted of exposing the mice to the apparatus for 5 min at a constant speed of 4 rpm. Mice that fall during the training session are placed back on the apparatus until the training session time has elapsed. An hour following the training session, mice are placed back on the rod for a 2-min session in which speed increases steadily over 2 min from 5 to 40 rpm. If a mouse does not fall during the 2 min, the trial ends at 2 min. Each animal was tested over 3 d and the latency to fall was averaged for each mouse. This assay was conducted in 21 control mice (14 males, seven females) and 18 *Drd2^Pet1-CKO^* (13 males, five females).

#### Acoustic startle response

Mice were placed in a perforated holder (acrylic cylinder with 3.2-cm internal diameter) that allowed movement to be monitored. Animal holders were placed on top of a transducer platform, measuring the active response to both weak and startle stimuli, adjacent to a speaker, within an individual acoustic chamber (Med Associates). Each session consisted of a 5 min acclimation period followed by 10 blocks of 11 trials each with white noise acoustic stimuli (20–120 dB). Each startle stimulus (20–120 dB, in 10-dB increments) was played once per block, in a quasi-random order with a variable intertrial interval of 10–20 s (average of 15 s). The duration of the stimulus was 40 ms. Responses were recorded for 150 ms from startle onset and are sampled every ms. Mice were placed back into the home cage immediately after testing. Males and female were run on different days. This assay was conducted in 30 control mice (14 males, 16 females) and 28 *Drd2^Pet1-CKO^* (13 males, 15 females), as two separate cohorts per sex.

#### Tube test of social dominance

Two age-matched (∼P90), weight-matched mice of the same sex are introduced into opposite ends of a clear PVC tube (30.5 cm in length with an internal diameter of 2.5 cm) allowing them to interact in the middle but not pass each other within the tube. The subordinate mouse will back out allowing the dominant mouse to pass through (Lindzey et al., 1961). For each pair, five consecutive trials were run with a maximum time of 2 min per trial. Trials ended when one mouse backed out of the tube such that all four limbs are outside of the tube which was then recorded as a “backout” for that mouse. Matches lasting >2 min were excluded from analysis and scored as a draw. Side of introduction to the tube were alternated between trials and the tube was cleaned with ethanol between each trial. Opponents were from different litters and had never been housed together. This assay was conducted in 24 *Drd2^Pet1-CKO^* males versus 24 control males and 23 *Drd2^Pet1^*^-CKO^ females versus 23 control females, conducted across three cohorts of animals.

#### Resident-intruder assay

*Drd2^Pet1^*^-CKO^ or control mice were group-housed with male siblings until adulthood (P90) when they were single-housed for one night in the test cage to establish territorial residency. On day 1, a five-week-old Swiss Webster (Charles River) male, the “intruder,” was introduced to the cage divided with a clear perforated divider for 5 min. After 5 min, the perforated divider was removed, and the mice could interact for 5 min, in which the encounter was video recorded. Number of attack bites were counted by a trained, blinded viewer. The intruder mouse was introduced for 3 d to obtain an average number of attack bites per day. The intruder mouse had a lower body weight than the resident male. This assay was conducted only in males, as female laboratory-reared mice do not display territorial aggression (Palanza, 2001; Lonstein and Gammie, 2002) This assay was conducted in 26 *Drd2^Pet1-CKO^* and 24 controls.

#### Auditory brainstem response (ABR)

ABRs were recorded in a separate cohort of adult mice (males: 10 control and seven *Drd2^Pet1-CKO^*; females: eight control and seven *Drd2^Pet1-CKO^*) aged P71–P102 to correspond to the age of animals in other assays. ABRs were conducted similar to (Maison et al., 2013). Mice were anesthetized by intraperitoneal injection of ketamine (100 mg/kg) and xylazine (7.5 mg/kg) and placed in a soundproof chamber on a heating pad. Acoustic stimuli were delivered using EPL Cochlear Function Test Suite (CFTS) software and analyzed using ABR peak analysis software [1.1.1.9, Massachusetts Eye and Ear (MEE)]. All ABR thresholds, amplitudes, and latencies were read by an investigator blind to mouse genotype.

### Electrophysiology

Slice preparation and whole-cell patch-clamp recordings were conducted as previously described (Rood et al., 2014; Niederkofler et al., 2016). Briefly, to assess membrane and action potential (AP) characteristics a protocol of repeated sweeps of 500-ms current injections stepping in 20-pA steps from −80 to 180 pA was administered to cells in current clamp. Data were analyzed using Clampfit (Molecular Devices). Some cells included in cell property analyses were also used to generate data on the function of DRD2 receptors in the DR (Niederkofler et al., 2016). However, the intrinsic cell properties data we present in this article have not been previously published and include cells not part of the Niederkofler et al. (2016) dataset.

### Projection mapping

Brain tissue from six females and five males from different litters, but with a female and male from the same litter where possible, were collected at P90 and processed as previously described (Niederkofler et al., 2016). Target region identification was based on anatomic landmarks identified by DAPI staining, anti-choline acetyltransferase (goat polyclonal anti-ChAT,1:500, AB144P; EMD234 Millipore) staining, and/or anti-tyrosine hydroxylase (rabbit anti-TH, 1:1000, AB152, Millipore) staining. Staining and imaging protocols were identical among the eleven samples analyzed.

### Quantification of target innervation

Target innervation was quantified in a similar manner to (Niederkofler et al., 2016). Briefly, image stacks were acquired bilaterally per brain region analyzed for each animal using a Nikon Ti inverted spinning disk microscope with a Plan Fluor 40×/1.3 Oil DIC H/N2 objective, 488-, 561-, and 647-nm laser lines, and Andor Zyla 4.2 Plus sCMOS monochrome camera. Images were acquired with Nikon Elements Acquisition software AR 5.02. Image stacks (.nd2 files) were imported to FIJI for analysis of axon projection area. Each stack contained 21 optical slices of 0.3 μm. Innervation density was quantified by a FIJI macro, such that all images, were treated identically, including background subtraction, thresholding and particle counting as described in (Niederkofler et al., 2016). We then divided the total area occupied by the projection signal by the total area of the 21 optical slices to obtain the percent area occupied by projection signal. This was then averaged within images of the same brain region across male or female samples. Brain regions analyzed were either those previously described to be innervated by *Drd2-Pet1* neurons in males only (Niederkofler et al., 2016) or those involved in auditory processing and ASR.

### Statistical analyses

Data are presented as mean ± SEM. Statistical analyses were conducted in GraphPad Prism version 8.1. Statistical significance was determined by unpaired *t* test between control versus *Drd2^Pet1^*^-CKO^ groups or male versus female groups except where noted: open field, forced swim test, acoustic startle response, and ABR statistical significance was determined using two-way ANOVA. For the resident-intruder assay, the tube test of social dominance, and rotarod, statistical significance was determined using the non-parametric Mann–Whitney *U* test. A result was considered significant if *p* < 0.05. Detailed statistical results are reported in Table 2.

**Table 2:** Statistical analysis

Behavior/experiment	Line	Data structure (normality)	Type of test	Power		
				Comparison	*F*/df	*p*
Validation of *Drd2* CKO	1*E*	Yes	Unpaired *t* test	Control vs *Drd2^Pet1-^*^CKO^	*t* = 4.514, df = 10	*p* = 0.0011
Open field distance	2*A*	Yes	Repeated-measures ANOVA	F1, genotype	*F*_(1,24)_ = 0.6405	*p* = 0.4314
				F2, time	*F*_(11,264)_ = 47.99	*p* < 0.0001
				(F1 × F2)	*F*_(11,264)_ = 0.8441	*p* = 0.5960
Open field % distance traveled	2*B*	Yes	Unpaired *t* test	Control vs *Drd2^Pet1-^*^CKO^	*t* = 1.781, df = 24	*p* = 0.0876
Rotarod	2*C*	No	Mann–Whitney, two-tailed	Control vs *Drd2^Pet1-^*^CKO^	M–W *U* = 142	*p* = 0.1899
Elevated plus maze (% time in open arm)	2*D*	Yes	Unpaired *t* test	Control vs *Drd2^Pet1-^*^CKO^	*t* = 1.250, df = 24	*p* = 0.2234
Tail suspension test	2*E*	Yes	Unpaired *t* test	Control vs *Drd2^Pet1-^*^CKO^	*t* = 0.3485, df = 24	*p* = 0.7305
Forced swim test	2*F*	Yes	Repeated-measures ANOVA	F1, genotype	*F*_(1,24)_ = 0.2678	*p* = 0.6095
			F2, time	*F*_(5,120)_ = 8.916	*p* < 0.0001
			(F1 × F2)	*F*_(5,120)_ = 0.3090	*p* = 0.9067
Contextual fear conditioning (baseline freezing)	2*G*	Yes	Unpaired *t* test	Control vs *Drd2^Pet1-^*^CKO^	*t* = 0.6682, df = 24	*p* = 0.5104
Contextual fear conditioning (test freezing)			Unpaired *t* test	Control vs *Drd2^Pet1-^*^CKO^	*t* = 0.0127, df = 24	*p* = 0.9900
Water T maze (%correct during acquisition)	2*H*	Yes	Repeated-measures ANOVA	F1, genotype	*F*_(1,24)_ = 0.08249	*p* = 0.7764
				F2, time	*F*_(4,89)_ = 50.12	*p* < 0.0001
				(F1 × F2)	*F*_(4,89)_ = 0.6698	*p* = 0.6147
Water T maze (%correct during reversal)		Yes	Repeated-measures ANOVA	F1, genotype	*F*_(1,24)_ = 0.1631	*p* = 0.6899
				F2, time	*F*_(4,96)_ = 172.4	*p* < 0.0001
				(F1 × F2)	*F*_(4,96)_ = 1.477	*p* = 0.2153
ASR (M)	3*C*	Yes	Repeated-measures ANOVA	F1, genotype	*F*_(1,25)_ = 0.0840	*p* = 0.7745
				F2, dB	*F*_(10,250)_ = 28.99	*p* < 0.0001
				(F1 × F2)	*F*_(10,250)_ = 0.3037	*p* = 0.9798
ASR habituation (M)	3*D*	Yes	Pearson *r* correlation	Control trial number × startle response	*r* = −0.195	*p* = 0.5893
				*Drd2^Pet1-CKO^* trial number × startle response	*r* = 0.136	*p* = 0.7079
ASR latency (M)	3*E*	Yes	Repeated-measures ANOVA	F1, genotype	*F*_(1,25)_ = 2.425	*p* = 0.1319
				F2, dB	*F*_(10,250)_ = 21.67	*p* < 0.0001
				(F1 × F2)	*F*_(10,250)_ = 0.4722	*p* = 0.9071
ASR (F)	3*F*	Yes	Repeated-measures ANOVA	F1, genotype	*F*_(1,29)_ = 13.26	*p* = 0.0011
				F2, dB	*F*_(10,29)_ = 35.29	*p* < 0.0001
				(F1 × F2)	*F*_(10,290)_ = 7.475	*p* < 0.0001
ASR habituation (F)	3*G*	Yes	Pearson *r* correlation	Control trial number × startle response	*r* = 0.1171	*p* = 0.7473
				*Drd2^Pet1-CKO^* trial number × startle response	*r* = 0.05165	*p* = 0.8873
ASR latency (F)	3*H*	Yes	Repeated-measures ANOVA	F1, genotype	*F*_(1,29)_ = 0.3748	*p* = 0.5452
				F2, dB	*F*_(10,290)_ = 20.59	*p* < 0.0001
				(F1 × F2)	*F*_(10,290)_ = 1.058	*p* = 0.3953
PPI (M)	3*I*	Yes	Repeated-measures ANOVA	F1, genotype	*F*_(1,12)_ = 0.6625	*p* = 0.4315
				F2, prepulse dB	*F*_(2,24)_ = 42.86	*p* < 0.0001
				(F1 × F2)	*F*_(2,24)_ = 4.104	*p* = 0.0293
PPI (F)	3*J*	Yes	Repeated-measures ANOVA	F1, genotype	*F*_(1,10)_ = 0.6526	*p* = 0.4380
				F2, prepulse dB	*F*_(2,20)_ = 31.34	*p* < 0.0001
				(F1 × F2)	*F*_(2,20)_ = 1.609	*p* = 0.2249
ABR amplitude (M)	4*C*	Yes	Repeated-measures ANOVA	F1, genotype	*F*_(1,15)_ = 1.770	*p* = 0.2032
				F2, peak	*F*_(2,30)_ = 59.09	*p* < 0.0001
				(F1 × F2)	*F*_(2,30)_ = 1.059	*p* = 0.3595
ABR latency (M)	4*D*	Yes	Repeated-measures ANOVA	F1, genotype	*F*_(1,15)_ = 3.515	*p* = 0.0804
				F2, peak	*F*_(2,30)_ = 1171	*p* < 0.0001
				(F1 × F2)	*F*_(2,30)_ = 3.121	*p* = 0.0587
ABR threshold (M)	4*E*					
5.6		Yes	Unpaired *t* test	Control vs *Drd2^Pet1-^*^CKO^	*t* = 0.9535, df = 14	*p* = 0.3565
8		Yes	Unpaired *t* test	Control vs *Drd2^Pet1-^*^CKO^	*t* = 1.894, df = 14	*p* = 0.0791
16		Yes	Unpaired *t* test	Control vs *Drd2^Pet1-^*^CKO^	*t* = 1.103, df = 14	*p* = 0.2887
32		Yes	Unpaired *t* test	Control vs *Drd2^Pet1-^*^CKO^	*t* = 2.129, df = 7	*p* = 0.0708
ABR amplitude (F)	4*F*	Yes	Repeated-measures ANOVA	F1, genotype	*F*_(1,13)_ = 2.489	*p* = 0.1387
				F2, peak	*F*_(2,26)_ = 72.52	*p* < 0.0001
				(F1 × F2)	*F*_(2,26)_ = 0.0487	*p* = 0.9525
ABR latency (F)	4*G*	Yes	Repeated-measures ANOVA	F1, genotype	*F*_(1,13)_ = 0.0053	*p* = 0.9430
				F2, peak	*F*_(2,26)_ = 4360	*p* < 0.0001
				(F1 × F2)	*F*_(2,26)_ = 0.0822	*p* = 0.9213
ABR threshold (F)	4*H*					
5.6		Yes	Unpaired *t* test	Control vs *Drd2^Pet1-^*^CKO^	*t* = 0.1566, df = 13	*p* = 0.8770
8		Yes	Unpaired *t* test	Control vs *Drd2^Pet1-^*^CKO^	*t* = 0.1592, df = 14	*p* = 0.8757
16		Yes	Unpaired *t* test	Control vs *Drd2^Pet1-^*^CKO^	*t* = 0.9600, df = 14	*p* = 0.3533
32		Yes	Unpaired *t* test	Control vs *Drd2^Pet1-^*^CKO^	*t* = 1.644, df = 9	*p* = 0.1346
Social interaction (M, %time with stranger)	5*A*	Yes	Unpaired *t* test	Control vs *Drd2^Pet1-^*^CKO^	*t* = 0.6283, df = 12	*p* = 0.5415
Social interaction (F, %time with stranger)	5*B*	Yes	Unpaired *t* test	Control vs *Drd2^Pet1-^*^CKO^	*t* = 0.9598, df = 10	*p* = 0.3598
Resident-intruder assay	5*C*	No	Mann–Whitney, two-tailed	Control vs *Drd2^Pet1-^*^CKO^	M–W *U* = 289.5	*p* = 0.6649
Tube test of social dominance	5*E*					
Male		No	Mann–Whitney, two-tailed	Control vs *Drd2^Pet1-^*^CKO^	M–W *U* = 166	*p* = 0.0065
Female		No	Mann–Whitney, two-tailed	Control vs *Drd2^Pet1-^*^CKO^	M–W *U* = 253	*p* = 0.8123
*Drd2-Pet1* neuron count	6*A*	Yes	Unpaired *t* test	Male vs female	*t* = 0.8160, df = 12	*p* = 0.4304
Soma size	6*C*	Yes	Unpaired *t* test	Male vs female	*t* = 1.021, df = 8	*p* = 0.3372
Gene expression	6*D*					
*Dmd*		Yes	Unpaired *t* test	Male vs female	*t* = 0.9581, df = 7	*p* = 0.3699
*Drd2*		Yes	Unpaired *t* test	Male vs female	*t* = 1.514, df = 8	*p* = 0.1686
*Gad2*		Yes	Unpaired *t* test	Male vs female	*t* = 2.498, df = 8	*p* = 0.0370
*Serpini1*		Yes	Unpaired *t* test	Male vs female	*t* = 1.459, df = 7	*p* = 0.1879
*%Gad2+ Drd2-Pet1* neurons	6*E*	Yes	Unpaired *t* test	Male vs female	*t* = 1.876, df = 8	*p* = 0.0975
% *Drd2*-Exon7/8+	7*B*	Yes	Unpaired *t* test	Control vs *Drd2^Pet1-^*^CKO^ with *Pet1* probe	*t* = 0.1291, df = 10	*p* = 0.8998
		Yes	One-way ANOVA	Control/*Pet1* probe vs *Drd2^Pet1-^*^CKO^/ *Pet1* probe vs *Drd2^Pet1^*^-CKO^ /Cre probe	*F*_(2,19)_ = 0.1003	*p* = 0.9051
% *Gad2* in Cre+ neurons	7*D*	Yes	Unpaired *t* test	Male vs female	*t* = 3.057, df = 8	*p* = 0.0157
*Gad2* punctae per cell	7*E*	Yes	Unpaired *t* test	Male vs female	*t* = 1.768, df = 8	*p* = 0.1151
Nucleus area	7*F*	Yes	Unpaired *t* test	Male vs female	*t* = 0.9931, df = 8	*p* = 0.3497
Resting membrane potential	8*A*	Yes	Unpaired *t* test	Male vs female	*t* = 0.2113, df = 61	*p* = 0.8334
Membrane resistance	8*B*	Yes	Unpaired *t* test	Male vs female	*t* = −0.4084, df = 61	*p* = 0.6844
AP threshold	8*B*	Yes	Unpaired *t* test	Male vs female	*t* = 1.8197, df = 61	*p* = 0.0737
AP amplitude	8*D*	Yes	Unpaired *t* test	Male vs female	*t* = −1.0474, df = 61	*p* = 0.2990
AP duration	8*E*	Yes	Unpaired *t* test	Male vs female	*t* = −2.2583, df = 61	*p* = 0.0275
AHP amplitude	8*F*	Yes	Unpaired *t* test	Male vs female	*t* = 1.350, df = 61	*p* = 0.1821
Innervation densities	9*C*					
DPGi		Yes	Unpaired *t* test	Male vs female	*t* = 1.285, df = 9	*p* = 0.2308
PAG		Yes	Unpaired *t* test	Male vs female	*t* = 0.2398, df = 9	*p* = 0.8158
mPOA		Yes	Unpaired *t* test	Male vs female	*t* = 0.1978, df = 9	*p* = 0.8476
DLG		Yes	Unpaired *t* test	Male vs female	*t* = 0.07798, df = 9	*p* = 0.9395
mHb		Yes	Unpaired *t* test	Male vs female	*t* = 0.6732, df = 9	*p* = 0.5178
PnC		Yes	Unpaired *t* test	Male vs female	*t* = 0.7901, df = 9	*p* = 0.4498
IC		Yes	Unpaired *t* test	Male vs female	*t* = 0.5350, df = 9	*p* = 0.6056
LL		Yes	Unpaired *t* test	Male vs female	*t* = 0.9100, df = 9	*p* = 0.3865
SOC		Yes	Unpaired *t* test	Male vs female	*t* = 0.9282, df = 9	*p* = 0.3775
CNC		Yes	Unpaired *t* test	Male vs female	*t* = 0.2997, df = 9	*p* = 0.7712

Statistical values are provided for behavioral analyses of *Drd2^Pet1^*^-CKO^ mice and comparison of *Drd2-Pet1* neuron properties in male versus female mice. Figure numbers are included to reference corresponding graphs. Statistical analyses were conducted in GraphPad Prism version 8.1.

## Results

### Visualization of *Drd2-Pet1* serotonergic neurons and the loss of *Drd2* gene expression in *Drd2^Pet1-CKO^* mice

**Figure 1.**
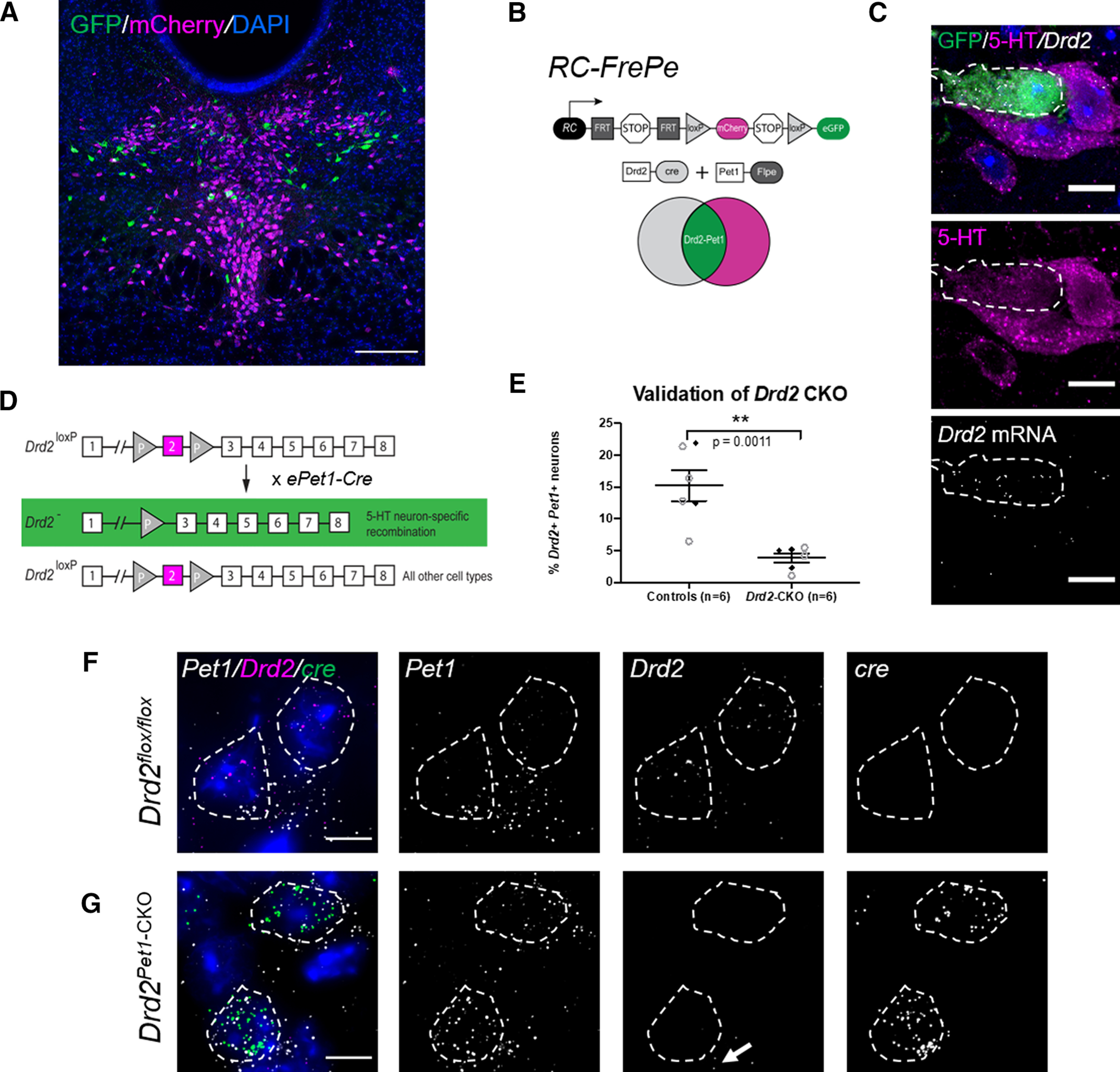
Visualization of *Drd2-Pet1* serotonergic neurons and the loss of *Drd2* gene expression in *Drd2^Pet1^*^-CKO^ mice. ***A***, *Drd2-Pet1* neurons are intersectionally labeled with GFP (green) and *Pet1*-only positive cell bodies labeled with mCherry (magenta) in a coronal brain section of the DR from a P90 triple transgenic *Drd2-Cre;Pet1-Flpe;RC:FrePe* mouse. Scale bars: 200 μm. ***B***, Intersectional genetic strategy: expression of *Drd2-Cre* and *Pet1-Flpe* transgenes results in dual recombination of intersectional allele, *RC:FrePe*, labeling cells expressing *Drd2* and *Pet1* with GFP. ***C***, Dual immunohistochemistry for GFP (green) and 5-HT (serotonin, magenta) coupled with FISH detection of *Drd2* mRNA, which shows co-localization of intersectionally labeled *Drd2-Pet1* neuron cell bodies with 5-HT and *Drd2* mRNA. Scale bars: 10 μm. ***D***, Strategy for conditional deletion of *Drd2* in serotonergic neurons (referred to throughout as *Drd2^Pet1^*^-CKO^). Cre recombination excises *Drd2* exon 2 (magenta) producing serotonergic-specific (boxed in green) deletion of *Drd2* gene sequences. ***E***, Percentage (mean ± SEM) of *Pet1*+ serotonergic neurons that express *Drd2* in control (*n* = 6) versus *Drd2^Pet1^*^-CKO^ (*n* = 6) shows reduction of *Drd2* expression in *Pet1*+ neurons (controls: 15.23 ± 2.41 *Drd2-Pet1* dual positive neurons per brain, *Drd2^Pet1^*^-CKO^: 3.87 ± 0.73 *Drd2-Pet1* dual positive neurons per brain, *p* = 0.0011, unpaired *t* test). Filled black diamonds represent male mice, open gray circles represent female mice. ***F***, ***G***, FISH on (***F***) control and (***G***) *Drd2^Pet1^*^-CKO^ tissue. *Drd2* transcripts detected in *Pet1*+ cells in control sections, but not in *Drd2^Pet1^*^-CKO^ mice, indicative of loss of *Drd2*. *cre* transcript is not present in control (***F***, far right) but is present in *Drd2^Pet1^*^-CKO^ *Pet1* cells, as expected (***G*,** far right). *Pet1*, *Drd2*, and *cre* transcript are shown separately in grayscale. Note *Drd2* expression remains in non-*Pet1* cells (arrow). Dotted lines drawn to encircle DAPI nuclei. Scale bars: 25 μm.

As our first step, we confirmed the anatomic distribution of *Drd2-Pet1* neurons in the mouse brainstem, observing cell soma distributed across the rostral and lateral regions of the DR nucleus (Fig. 1*A*) as previously reported (Niederkofler et al., 2016). *Drd2-Pet1* cells were marked by GFP expression in triple transgenic *Drd2-Cre;Pet1-Flpe;RC-Frepe* (Gong et al., 2007; Jensen et al., 2008; Brust et al., 2014) mice in which cells positive for both Cre and Flpe activity – here those cells having expressed *Drd2* and *Pet1* – have recombined the *RC-FrePe* intersectional reporter allowing GFP expression; Flpe recombination alone configured *RC-FrePe* to drive mCherry expression, thus marking the remaining *Pet1+* (*Drd2*-negative) serotonergic neurons (Fig. 1*A*,*B*). As expected (Niederkofler et al., 2016), GFP+ *Drd2-Pet1* neurons showed detectable 5-HT by immunostaining and *Drd2* mRNA by fluorescent *in situ* hybridization (FISH) (Fig. 1*C*).

To query the behavioral requirement for *Drd2* gene expression in *Drd2-Pet1* neurons, we deployed the e*Pet-cre* driver (Scott et al., 2005) to delete floxed *Drd2* gene sequences (Bello et al., 2011), creating a functional null *Drd2* allele selectively in *Pet1* neurons (Fig. 1*D*), and then subjected these *Drd2^Pet1^*^-CKO^ mice to behavioral phenotyping. Cre-negative, *Drd2^flox/flox^*littermates served as controls. To confirm loss of *Drd2* gene expression in *Pet1* neurons, we analyzed *Drd2^Pet1^*^-CKO^ and control *Drd2^flox/flox^* brain tissue sections by mRNA *in situ* hybridization using a probe designed to detect exon 2-containing *Drd2* mRNA, as exon 2 was the floxed gene portion to be excised by Cre recombination; concomitant identification of serotonergic neurons was by detection of *Pet1* transcripts (Fig. 1*F*,*G*). Robust loss of *Drd2* expression was observed in serotonergic neurons in both male and female mice [15.23 ± 2.41% of *Pet1*+ neurons in the DR express *Drd2* transcripts in controls (*n* = 6), consistent with prior findings, compared with 3.87 ± 0.73% in *Drd2^Pet1^*^-CKO^s (*n* = 6), *p* = 0.0011, unpaired *t* test; Fig. 1*E*]. The few residual *Pet1+* cells harboring *Drd2* transcripts likely reflects a limitation in cell capture by the *ePet-cre* driver. Reliable immunodetection to confirm the expected parallel loss of DRD2 protein in PET1 cells remains unavailable.

### Behavioral assessments in *Drd2^Pet1-CKO^* mice and the detection of sex-specific sensory, defensive, and social behaviors

**Figure 2.**
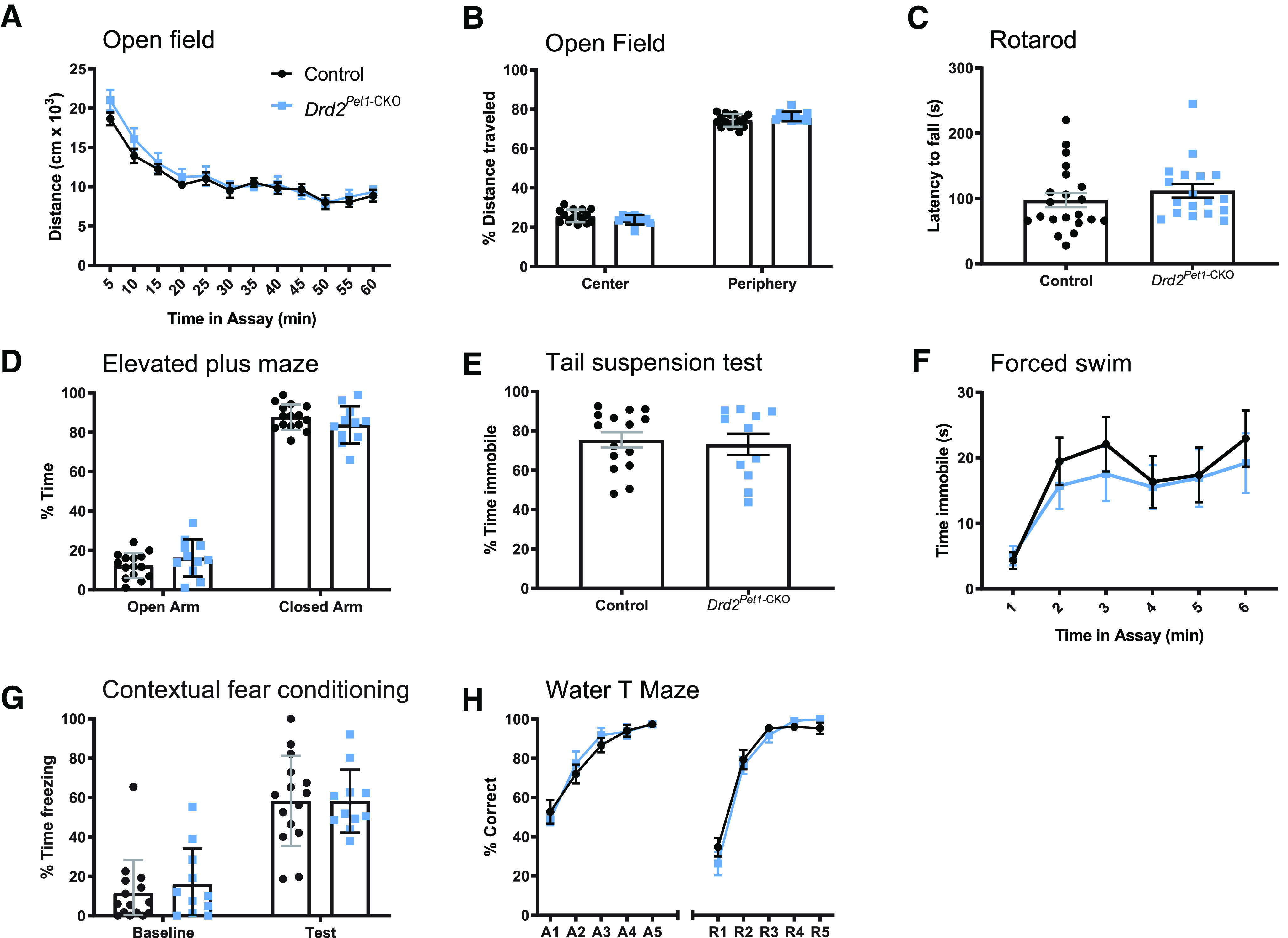
*Drd2^Pet1^*^-CKO^ mice are largely behaviorally normal. *Drd2^Pet1^*^-CKO^ (blue symbols) mice show behaviors indistinguishable from controls (black symbols) in measures of locomotion: (***A***, ***B***) open field test and (***C***) rotarod; measures of anxiety-like and depression-like behavior: (***D***) elevated plus maze, (***E***) tail suspension test, and (***F***) forced swim test; or learning and memory: (***G***) contextual fear conditioning and (***H***) water T maze; *n* = 15 control mice (8 males, 7 females) and 11 *Drd2^Pet1^*^-CKO^ (6 males, 5 females), except for ***C*** where, *n* = 21 control mice (14 males, 7 females) and 18 *Drd2^Pet1^*^-CKO^ (13 males, 5 females). Each symbol represents one animal, error bars represent SEM. No significant differences (*p* > 0.05) between *Drd2^Pet1^*^-CKO^ and controls were observed. No sex-specific (male vs female) phenotypes observed. For assay details, see Materials and Methods; for statistical details, see Table 2.

Having validated effective loss of *Drd2* expression specific to *Pet1* neurons in *Drd2^Pet1-^*^CKO^ mice, next, we screened these mice for behavioral alterations in comparison to sibling control *Drd2^flox/flox^* (Cre-negative) mice. Locomotor behaviors were explored first because they are known to be influenced by serotonergic and dopaminergic manipulations (Baik et al., 1995; Gainetdinov et al., 1999; Holmes et al., 2003; Seo et al., 2019), and because motor alterations can affect performance in and interpretation of subsequent behavioral assays. Notably, we found no differences between *Drd2^Pet1^*^-CKO^ versus control mice (males or females) in the locomotor behaviors reflected in the open field and rotarod tests, such as distance traversed (Fig. 2*A*) and location within the field (Fig. 2*B*), vertical rearing, length of time on the rotating rod (Fig. 2*C*), which reflects balance, coordination, physical conditioning, and motor-planning. Next, we explored measures of depression-like and anxiety-like behaviors, as they are altered in various 5-HT-pathway or DA-pathway mouse models and pharmacological manipulation of these neurotransmitter systems show positive clinical effect. (Lucki, 1998; Hendricks et al., 2003; Holmes et al., 2003; Grace, 2016). We observed no differences in performance in the elevated plus maze (Fig. 2*D*), tail suspension test (Fig. 2*E*), or forced swim test (Fig. 2*F*) in *Drd2^Pet1^*^-CKO^ males and females compared with littermate controls. Additionally, contextual fear conditioning (Fig. 2*G*) and water T-maze acquisition and reversal (Fig. 2*H*) were not affected, suggesting no impairment of memory and learning in *Drd2^Pet1-CKO^* mice.

**Figure 3.**
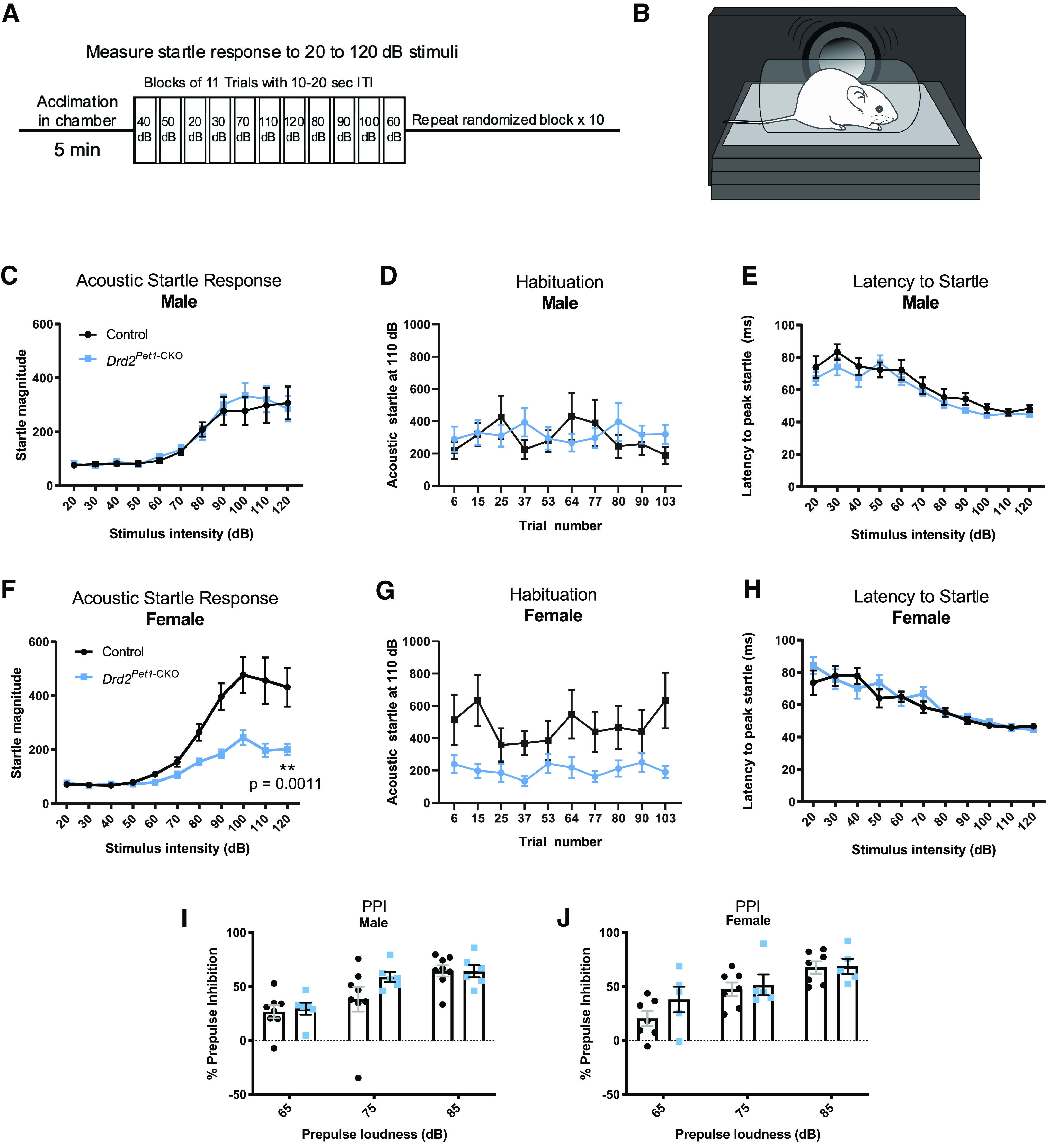
*Drd2^Pet1^*^-CKO^ females, but not males, display attenuated acoustic startle responses (ASR). ***A***, Schematic of ASR experimental design. After an initial 5-min acclimation, mice are exposed to 10 blocks of 11 trials of auditory stimuli ranging from 20 to 120 dB in quasi-randomized order with a 10- to 20-s intertrial interval (ITI). ***B***, Schematic of ASR measurement apparatus, mouse is placed in a perforated holding chamber atop transducer platform adjacent to speaker (for detailed description, see Materials and Methods). ***C***, ***F***, Averaged ASR magnitudes (mean ± SEM) across increasing stimulus intensities in (***C***) male *Drd2^Pet1^*^-CKO^ (blue, *n* = 13) and controls (black, *n* = 14), no significant difference, *p* = 0.7745, two-way ANOVA and (***F***) female *Drd2^Pet1^*^-CKO^ (blue, *n* = 15) and controls (black, *n* = 16), *Drd2^Pet1^*^-CKO^ females display significantly attenuated ASR, *p* = 0.0011, two-way ANOVA. ***D***, ***G***, Group averaged ASR for 10 trials at 110-dB stimulus in (***D***) males and (***G***) females, demonstrates no habituation to the startle stimulus; *x*-axis numbers refers to trial number out of 110 total trials. ***E***, ***H***, No significant differences in latency to startle are observed in (***E***) males, *p* = 0.1319, two-way ANOVA and (***H***) females, *p* = 0.5452, two-way ANOVA. ***I***, ***J***, No significant differences in prepulse inhibition of acoustic startle are observed in (***I***) males (*n* = 8 control, 6 *Drd2^Pet1^*^-CKO^), *p* = 0.4325, two-way ANOVA or (***J***) females (*n* = 7 control, 5 *Drd2^Pet1^*^-CKO^, *p* = 0.4380, two-way ANOVA).

Because the serotonergic and dopaminergic systems are implicated in modulating the ASR (Davis and Aghajanian, 1976; Davis et al., 1980; Meloni and Davis, 1999, 2000a,b), we explored that next. The ASR is an evolutionarily conserved reflex involving rapid contraction of facial and skeletal muscles into a protective posture in response to a loud, threatening stimulus. We hypothesized that *Drd2-Pet1* neurons modulate this response, given their dense projections to auditory brain regions (Niederkofler et al., 2016) and the observation that following acoustic startle, the activity of certain serotonergic neurons increases in the lateral wings of the DR (Spannuth et al., 2011), a location in which we find *Drd2-Pet1* neurons. We measured startle responses to weak and startling stimuli ranging from 20 to 120 dB presented in a randomized order (Fig. 3*A*,*B*). Female *Drd2^Pet1-CKO^* mice showed a significant decrease in ASR magnitude in response to startle stimuli (*n* = 15 *Drd2^Pet1-CKO^*, *n* = 16 control littermates, *p* = 0.0011, two-way ANOVA; Fig. 3*F*). By contrast, the male *Drd2^Pet1-CKO^* cohort was indistinguishable from their male littermate controls (*n* = 13 *Drd2^Pet1-CKO^*, *n* = 14 control littermates, *p* = 0.7745, two-way ANOVA; Fig. 3*C*). To prevent habituation to the startle stimuli, the different stimulus intensities were presented in a quasi-random order with varied intertrial intervals (see Materials and Methods), and indeed, startle responses in late as compared with early trials were indistinguishable (shown at 110 dB, trial number is not significantly correlated with startle magnitude, males: controls, *r* = −0.1950 and *Drd2^Pet1-CKO^*, *r* = 0.1360; females: controls, *r* = 0.1171 and *Drd2^Pet1-CKO^*, *r* = 0.0517, Pearson correlation; Fig. 3*D*,*G*). Further, we observed no differences in latency to startle in either females or males (Fig. 3*E*,*H*). Females were of similar mass (controls: 32.117 ± 3.15 g vs *Drd2^Pet1-CKO^*: 37.2 ± 2.427 g, unpaired *t* test, *p* = 0.2031) regardless of genotype, thus differences in weight and its relative impact on transduction of the startle response via the piezoelectric platform were not a confound.

While *Drd2^Pet1-CKO^* females showed diminished response magnitudes to startling acoustic stimuli, they nevertheless expressed normal acoustic prepulse inhibition (PPI) whereby even the diminished response to startling acoustic stimuli (e.g., 120-dB stimuli) was further blunted proportionately when immediately preceded by a weak, non-startling stimulus (e.g., 65-, 75-, or 85-dB stimuli; Fig. 3*I*,*J*). Thus, sensorimotor gating, as measured by acoustic PPI, appeared relatively intact; the acoustic dysfunction instead centered on the ASR itself.

**Figure 4.**
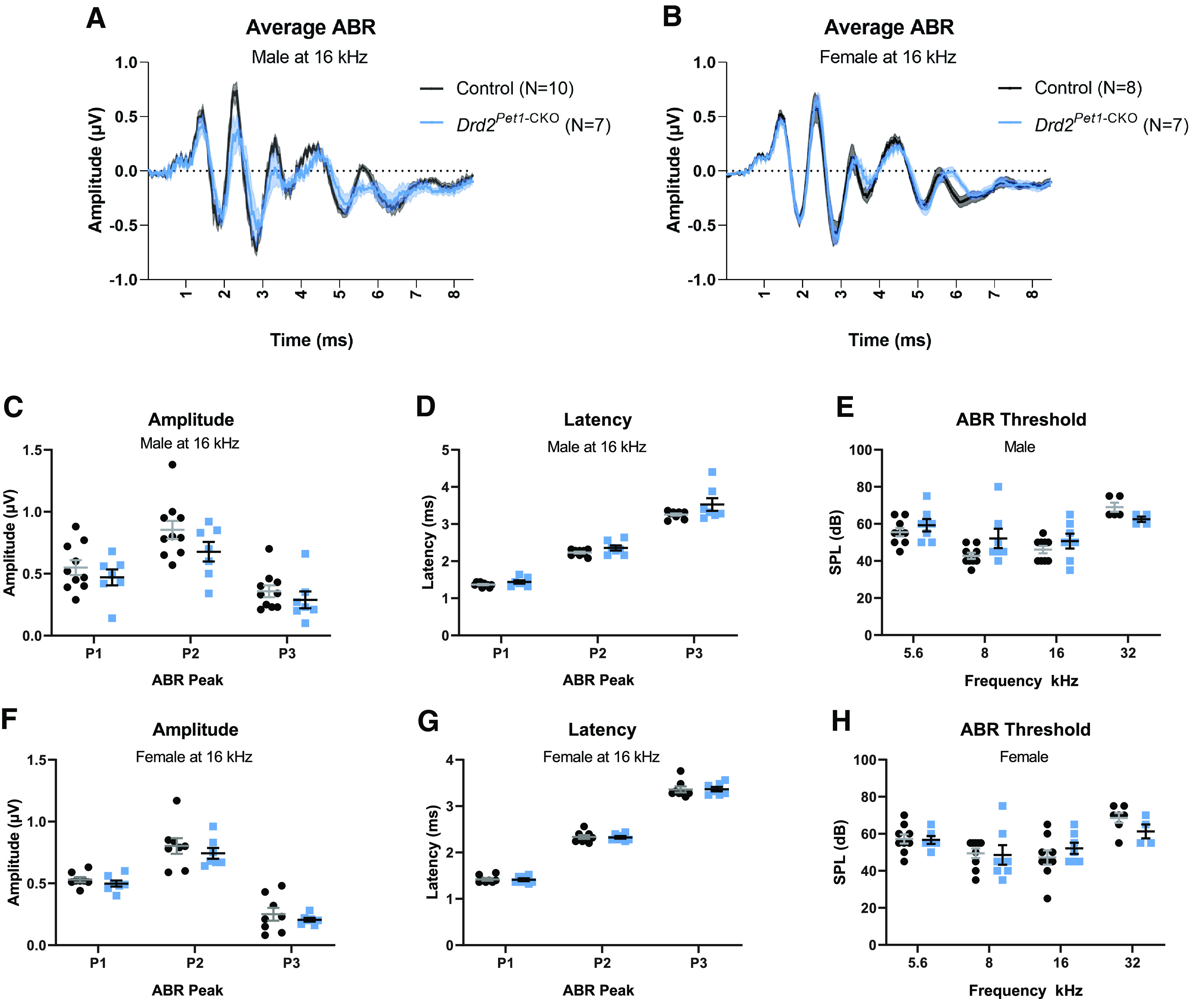
*Drd2^Pet1^*^-CKO^ mice show normal auditory responses. ***A***, ***B***, Average ABR waveforms at 16 kHz for (***A***) control (black, *n* = 10) and *Drd2^Pet1^*^-CKO^ (blue, *n* = 7) males and (***B***) for control (black, *n* = 8) and *Drd2^Pet1^*^-CKO^ (blue, *n* = 7) females. Average is shown by darker lines and shaded area shows SEM. ***C***, ***F***, ABR amplitudes for control (black) and *Drd2^Pet1^*^-CKO^ (blue; ***C***) male and (***F***) female mice for ABR peaks 1 through 3. No significant difference was observed between control and *Drd2^Pet1^*^-CKO^: males, *p* = 0.2032; females, *p* = 0.1387, two-way ANOVA. ***D***, ***F***, Latencies for control (black) and *Drd2^Pet1^*^-CKO^ (blue; ***D***) male and (***G***) female mice for ABR peaks 1 through 3. No significant difference was observed between control and *Drd2^Pet1^*^-CKO^: males, *p* = 0.0804; females, *p* = 0.9430, two-way ANOVA. Amplitudes and latencies shown at 80-dB SPL. ***E***, ***H***, ABR thresholds for control (black) and *Drd2^Pet1^*^-CKO^ (blue; ***E***) male and (***H***) female mice across frequencies tested (5.6, 8, 16, and 32 kHz). No significant difference was observed between control and *Drd2^Pet1^*^-CKO^ mice, *p* > 0.05 at all frequencies, unpaired *t* test.

Having observed attenuation of the ASR in female *Drd2^Pet1^*^-CKO^ mice, we assessed whether hearing was broadly disrupted as revealed by ABRs evoked by sound stimuli (Zhou et al., 2006). ABRs were recorded in response to pure tone stimuli at 5.6, 8, 16, and 32 kHz (*n* = 8 control females, 7 *Drd2^Pet1^*^-CKO^ females, and 10 control males, 7 *Drd2^Pet1-CKO^* males). Across all these frequencies, the measured ABR waveforms (averaged ABR waveforms shown at 16 kHz at 80-dB SPL; Fig. 4*A*,*B*), peak amplitudes [shown for peaks 1–3 at 16 kHz at 80-dB SPL for males (*p* = 0.2032, two-way ANOVA) and females (*p* = 0.1387, two-way ANOVA); Fig. 4*C*,*F*], and latencies to peaks [shown for peaks 1–3 at 16 kHz at 80-dB SPL for males (*p* = 0.0804, two-way ANOVA) and females (*p* = 0.9430, two-way ANOVA); Fig. 4*D*,*G*] were indistinguishable between *Drd2^Pet1^*^-CKO^ mice and littermate controls. As well, the ABR threshold to elicit a waveform was not significantly different between *Drd2^Pet1^*^-CKO^ and control mice at 5.6, 8, 16, or 32 kHz (*p* > 0.05 at all frequencies, unpaired *t* test) in males (Fig. 4*E*) or females (Fig. 4*H*). Thus, hearing overall, as measured by ABR, appeared largely unaffected in *Drd2^Pet1^*^-CKO^ mice.

ABRs were conducted in adult mice (ages P71–P102) to align with the age at which the other behavioral assays were performed. However at such ages, C57BL/6 mice, the strain background here, exhibit some age-related hearing loss at higher frequencies (Kane et al., 2012), which we saw here at 32 kHz with two control and three *Drd2^Pet1^*^-CKO^ females and five control and three *Drd2^Pet1^*^-CKO^ males. At all other tested frequencies, the ABRs were effectively normal for both genotypes, with one exception being a *Drd2^Pet1^*^-CKO^ female that exhibited undetectable ABRs at 5.6 kHz, but otherwise normal responses at all other frequencies tested including 32 kHz. These findings at 32 and 5.6 kHz are likely independent of the ASR phenotype observed in females because all animals had normal hearing at 8 and 16 kHz, frequencies included in the white noise startle stimulus of the ASR test.

**Figure 5.**
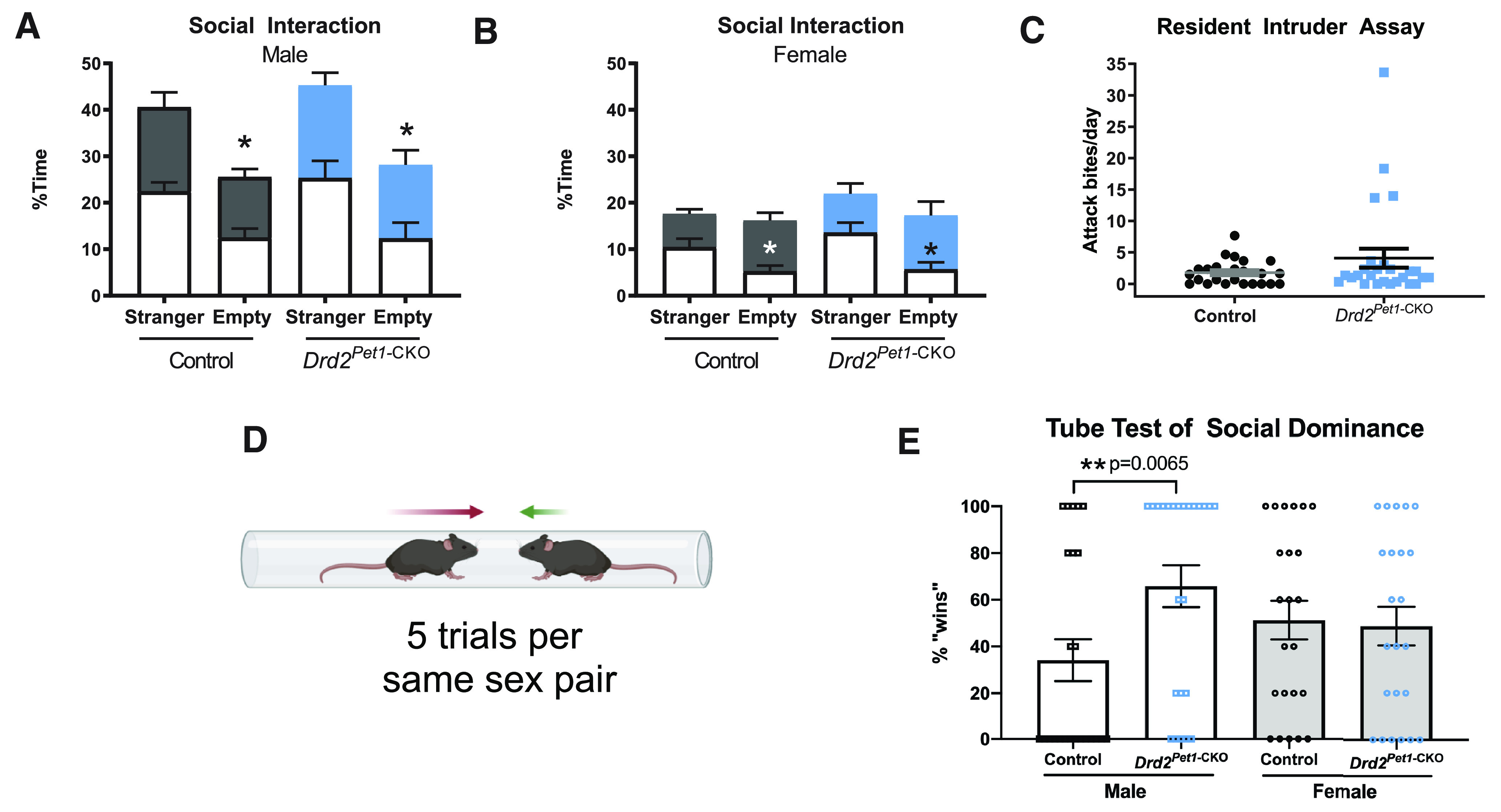
*Drd2^Pet1^*^-CKO^ males, but not females, display increased social dominance. ***A***, ***B***, Three chambered social interaction assay. No significant difference in time spent investigating a stranger mouse or an empty holder for (***A***) males (*n* = 8 controls compared with 6 *Drd2^Pet1^*^-CKO^, *p* = 0.541, unpaired *t* test) and (***B***) females (*n* = 7 controls compared with *n* = 5 *Drd2^Pet1^*^-CKO^, *p* = 0.358, unpaired *t* test). Investigation time is binned into 5-min intervals where white bars indicate first 5 min of assay and colored bars indicate last 5 min of assay. As expected, mice of both genotypes spent significantly less time investigating the empty holder than the stranger mouse noting that females of both genotypes only did so during the first 5 min of the assay. ***C***, Resident intruder assay of aggression. No significant difference in the average attack bites per day delivered to a Swiss Webster intruder mouse was observed between *Drd2^Pet1^*^-CKO^ males (*n* = 26, 4.07 ± 1.50 bites) aggression levels were not significantly different from controls (*n* = 24, 1.77 ± 0.39 bites; Mann–Whitney, two-tailed, *U* = 289.5, *p* = 0.6649). ***D***, Schematic of tube test (for details of assay, see Materials and Methods). Schematic created with BioRender. ***E***, *Drd2^Pet1^*^-CKO^ males (*n* = 24) demonstrate more dominance behavior than controls (*n* = 24) as they displayed increased winning in the tube test (controls: 34.17 ± 9% wins, *Drd2^Pet1^*^-CKO^: 65.83 ± 9% wins, *p* = 0.0065, Mann–Whitney, two-tailed, *U* = 166). Female *Drd2^Pet1^*^-CKO^ (*n* = 23) showed no difference in social dominance compared with controls (*n* = 23; controls: 51.3 ± 8%, *Drd2^Pet1^*^-CKO^: 48.7 ± 8% wins, *p* = 0.8123 Mann–Whitney, two-tailed, *U* = 253).

Next, we examined social behavior in *Drd2^Pet1^*^-CKO^ mice using the three-chambered test of sociability (Moy et al., 2004) that measures preference to investigate a social stimulus (a novel “stranger” mouse inside a holder) as compared with an object (an empty holder). *Drd2^Pet1-CKO^* mice showed no alterations in sociability compared with controls and both control and *Drd2^Pet1-CKO^* spent significantly more time investigating the stranger than the object (Fig. 5*A*,*B*). Females of both genotypes displayed preference toward the social stimuli only for the first 5 min of the assay (Fig. 5*B*, white bars), while males displayed this preference throughout the 10-min assay. Similar sex differences in sustained preference for the social stimulus have been described in C57BL/6J mice (Netser et al., 2017).

We assayed intermale, territorial aggression in a separate cohort of mice using a resident-intruder assay. Females were not tested, as they have been shown to display low or no aggression in most forms of this assay (Palanza, 2001; Lonstein and Gammie, 2002). We observed no statistically significant difference in number of attack bites delivered to the intruder mouse by *Drd2^Pet1-CKO^* males (*n* = 26) compared with number of attack bites delivered to the intruder by controls (*n* = 24; *Drd2^Pet1^*^-CKO^: 4.07 ± 1.50 bites, controls: 1.77 ± 0.39 bites, *p* = 0.6649, Mann–Whitney test; Fig. 5*C*) noting, however, that four *Drd2^Pet1^*^-CKO^ males displayed high levels of aggression.

To assay social dominance, we performed the tube test, which has relevance in females as well as males (Lindzey et al., 1961; van den Berg et al., 2015; Zhou et al., 2017). Two mice are simultaneously released into opposite ends of a clear tube of sufficiently narrow diameter that prevents mice from passing by each other and instead requires that one back out for the other, more dominant “winning” mouse, to move forward (Fig. 5*D*). *Drd2^Pet1^*^-CKO^ males won a higher percentage of trials against non-sibling, weight-matched, and genetic background-matched opponent males (shown as percent of trials won, *Drd2^Pet1^*^-CKO^: 65.83 ± 9%, *n* = 24; controls: 34.17 ± 9%, *n* = 24; *p* = 0.0065, Mann–Whitney test; Fig. 5*E*). By contrast, we observed no difference in percent of trials won by female *Drd2^Pet1^*^-CKO^ mice as compared with female sibling controls (*Drd2^Pet1^*^-CKO^: 48.7 ± 8%, *n* = 23; controls 51.3 ± 8%, *n* = 23; *p* = 0.8123, Mann–Whitney test; Fig. 5*E*).

### *Drd2-Pet1* neurons in males versus females exhibit differences in candidate molecular and biophysical properties but not in cell number

**Figure 6.**
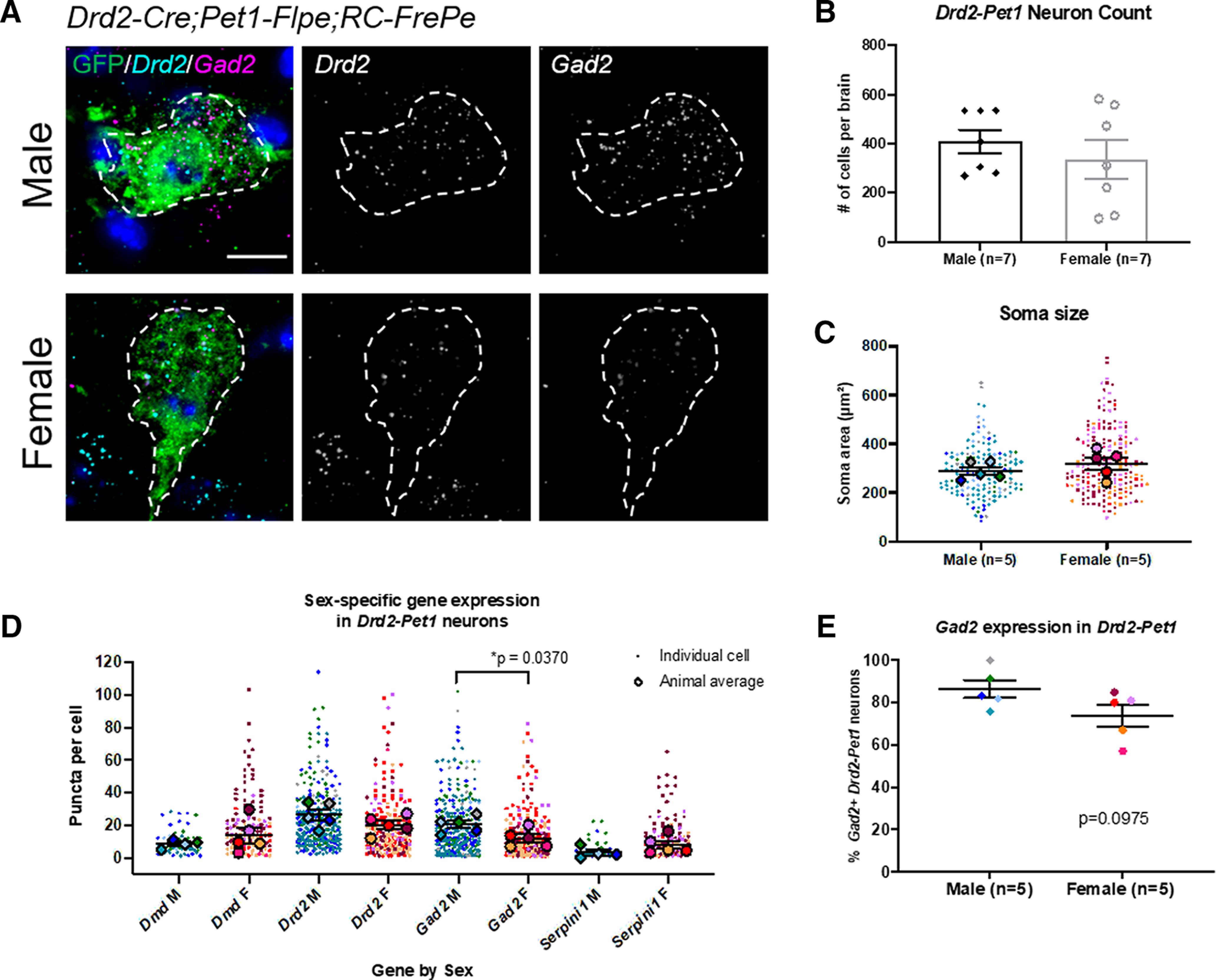
Sex-specific transcript level differences in *Drd2-Pet1* neurons. ***A***, Dual immunohistochemistry and FISH depicting green GFP*+ Drd2-Pet1* neurons along with transcript puncta in male (top) and female (bottom) brain sections from *Drd2-Cre;Pet1-Flpe;RC-FrePe* mice. *Drd2* (cyan) and *Gad2* (magenta) expression shown together and separately in gray scale. Scale bar: 10 μm. ***B***, Number of *Drd2-Pet1* neurons (GFP-positive cells in *Drd2-Cre;Pet1-Flpe;RC-FrePE* mice) per animal in males (black diamonds, *n* = 7) and females (open gray circles, *n* = 7) is not significantly different. Males: 410.40 ± 55.30 cells/brain, females: 313 ± 87.52 cells/brain, *p* = 0.4336, unpaired *t* test. ***C***, *Drd2-Pet1* neuron soma size (GFP+ cell body) does not differ in males (*n* = 5 males) versus females (*n* = 5 females), *p* = 0.3372, unpaired *t* test. ***D***, Number of FISH mRNA puncta per cell in males versus females. Male cells have significantly more *Gad2* puncta than female cells [20.46 ± 2.243 in males (*n* = 5) vs 12.20 ± 2.427 in females (*n* = 5), *p* = 0.0370, unpaired *t* test]. ***E*,** 86.47 ± 4.181% of male *Drd2-Pet1* cells express *Gad2* versus female 74.00 ± 5.168% in female cells, *p* = 0.0975, unpaired *t* test. Error bars indicate SEM throughout. For ***C***, ***D***, larger symbols outlined in black represent animal averages used for statistical analysis, smaller symbols represent individual cells, matched in color to the average.

Given these sex-specific differences in behaviors observed in *Drd2^Pet1^*^-CKO^ mice, next we looked for sex-specific differences in *Drd2-Pet1* cellular properties, beginning with cell number. Analyzing triple transgenic *Drd2-Cre; Pet1-Flpe; RC-FrePe* males versus females, we found no difference in number of GFP^+^ *Drd2-Pet1* neurons per brain (males: 410.40 ± 55.30 cells/brain, females: 313 ± 87.52 cells/brain, *p* = 0.4304, unpaired *t* test; Fig. 6*A*). Further, in both males and females, *Drd2-Pet1* neurons distributed as expected across the rostral-caudal and medial-lateral axis of the DR.

To understand whether gene expression might differ between male and female *Drd2-Pet1* neurons, we examined single-cell RNA sequencing data previously analyzed for expression of serotonergic pathway genes as validation that *Drd2-Pet1* cells were indeed serotonergic (Niederkofler et al., 2016). Comparison across sex, albeit lacking statistical significance given the small sample size, highlighted four genes for further evaluation, *Drd2*, *Dmd* (encoding Dystrophin, a component of protein scaffolds in the CNS; Perronnet and Vaillend, 2010), *Gad2* (encoding glutamate decarboxylase 2 involved in catalyzing the production of the neurotransmitter GABA), and *Serpini1* (encoding the serine protease Neuroserpin, important for synapse formation and plasticity; Galliciotti and Sonderegger, 2006). Quantitative *in situ* mRNA detection using dual FISH with immunodetection on tissue sections from *Drd2-Cre;Pet1-Flpe;RC-FrePe* mice revealed greater abundance of average *Gad2* transcripts (puncta) per cell in males versus females [*Gad2*: 20.46 ± 2.243 in males (*n* = 5) vs 12.20 ± 2.427 in females (*n* = 5), *p* = 0.0370, unpaired *t* test; Fig. 6*D*]. There was no difference in the percentage of *Drd2-Pet1* neurons expressing *Gad2* in male versus female mice (Fig. 6*E*). No difference in soma size (GFP-stained cell body) was observed between males and females suggesting that transcript differences were not because of larger soma volume measured (Fig. 6*C*). No significant differences in mRNA abundance were observed between males and females for *Dmd*, *Drd2*, or *Serpini1* (see Table 2).

**Figure 7.**
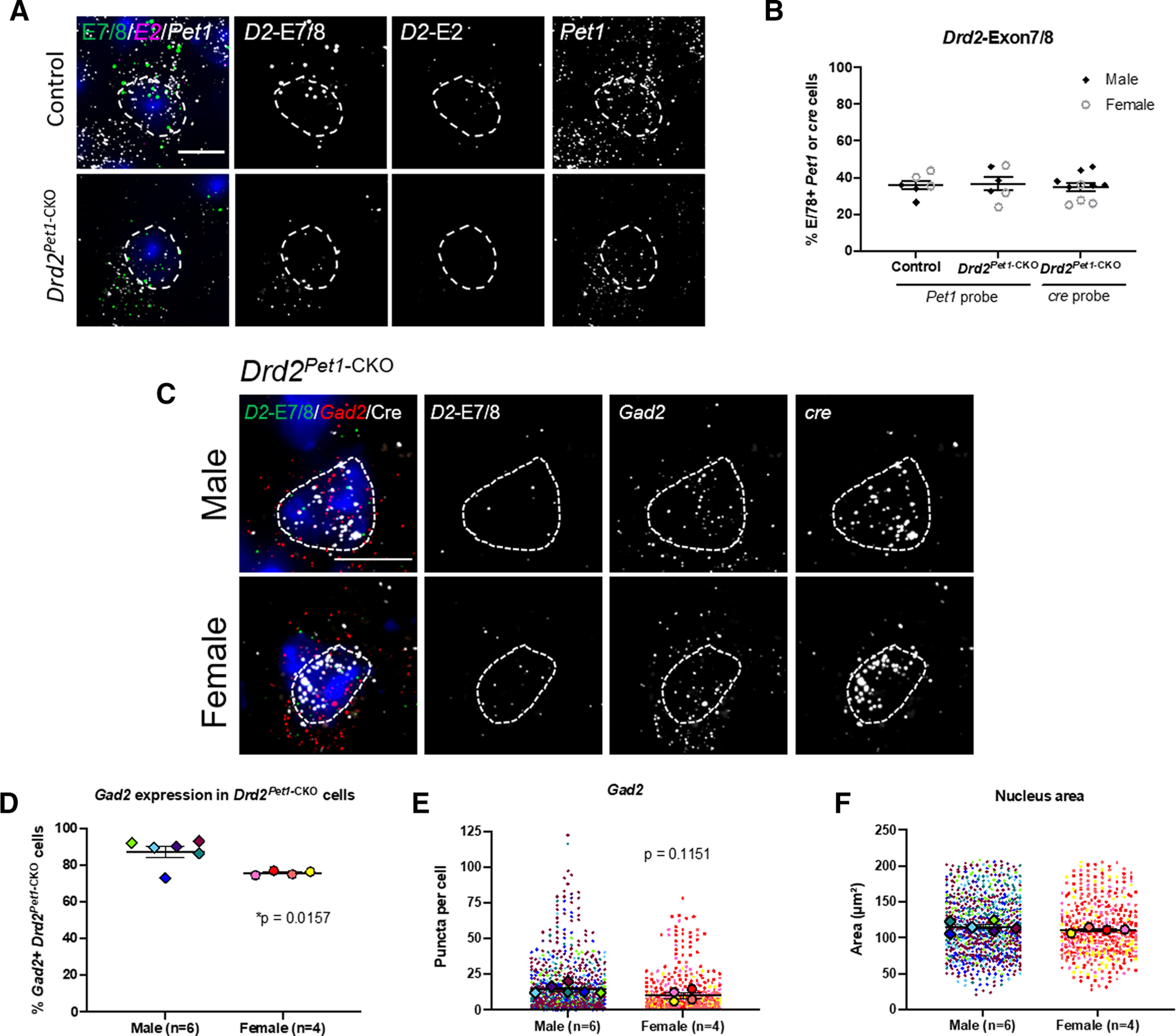
*Gad2* expression in *Drd2^Pet1-^*^CKO^ cells. ***A***, FISH with probes to *Drd2* exon 7/8 (*D2-*E7/8, green) and *Drd2* exon 2 (*D2-*E2, magenta) in *Pet1* (white) cells in control (top) and *Drd2^Pet1-^*^CKO^ (bottom) DR tissue. *D2-*E7/8, *D2-*E2, and *Pet1* expression shown together and separately in gray scale. ***B***, Percent of *Pet1+* cells (left and middle) with *Drd2*-Exon7/8 expression in control (35.97 ± 2.403%, *n* = 6) and *Drd2^Pet1^*^-CKO^ (36.53 ± 3.621%, *n* = 6), *p* = 0.8998, unpaired *t* test. Data also shown for percent of *cre* cells (right) with *Drd2-*Exon7/8, 34.91 ± 2.238%, compared with *Pet1* probe control and *Drd2^Pet1^*^-CKO^ *p* = 0.9051, one-way ANOVA. Males, black diamonds, females, open gray circles. ***C***, FISH showing *cre*+ *Drd2^Pet1^*^-CKO^ cells (white) with *Drd2-Exon7/8* (green) *and Gad2* (red) in male (top) and female (bottom) in the DR nucleus. *Drd2-Exon7/8*, *Gad2*, and *cre* are shown together and separately in gray scale. Scale bar: 10 μm. ***D***, A larger percentage of male *Drd2^Pet1^*^-CKO^ cells (87.44 ± 3.034%) express *Gad2* versus female *Drd2^Pet1^*^-CKO^ cells (75.76 ± 0.5862%), *p = 0.0157, unpaired *t* test. ***E***, Number of *Gad2* mRNA puncta per cell in *Drd2^Pet1^*^-CKO^ cells in males (*n* = 6) versus females (*n* = 4). Male cells have 14.25 ± 1.325 *Gad2* puncta per cell compared with 10.13 ± 2.074 in female cells, *p* = 0.1151, unpaired *t* test. ***F***, *Drd2^Pet1^*^-CKO^ nucleus size (area used to quantify puncta levels) does not differ in males (*n* = 6 males) versus females (*n* = 4 females), *p* = 0.3497, unpaired *t* test. Error bars indicate SEM throughout. For ***E***, ***F***, larger symbols outlined in black represent animal averages used for statistical analysis, smaller symbols represent individual cells, matched in color to the average.

As a first step toward understanding whether sex-specific gene expression differences observed in wild-type mice persist or are altered in *Drd2^Pet1^*^-CKO^ mice, we assessed *Gad2* transcript levels in *Drd2^Pet1^*^-CKO^ cells. In these cells, the floxed exon 2 of *Drd2* is excised by Cre recombination. Therefore, to identify mutant *Drd2* mRNA and thus the mutant *Drd2^Pet1^*^-CKO^ cells, we used a multi-probe strategy involving one probe to intact downstream exons 7 and 8 (referred to here as *Drd2-*E7/8), another to exon 2 (referred to as *Drd2*-E2), and another to either *cre* or *Pet1*. We examined expression in the DR region most enriched with *Drd2-Pet1* neurons. We found *Drd2-*E7/8+ puncta in *Pet1+* cells in both controls and *Drd2^Pet1^*^-CKO^ mice, whereas *Drd2-*E2+ puncta were detectable in control tissue but greatly reduced in *Drd2^Pet1^*^-CKO^ as expected given the efficiency of Cre-mediated gene deletion (Fig. 7*A*; see *Drd2-*E2 quantification in Fig. 1*E*). *Drd2-*E7/8 puncta were detected in 35.97 ± 2.403% of *Pet1*+ cells in control mice (*n* = 6) compared with 36.53 ± 3.621% of *Pet1*+ cells in *Drd2^Pet1^*^-CKO^ mice (*n* = 6; *p* = 0.8998, unpaired *t* test; Fig. 7*B*). Similarly, in a separate experiment using an *in situ* probe to *cre* mRNA, 34.91 ± 2.238% of *cre+* cells expressed *Drd2-*E7/8 (*n* = 10 mice, one-way ANOVA compared with *Pet1* control and *Drd2^Pet1^*^-CKO^ cell expression, *p* = 0.9051; Fig. 7*B*). Next, we analyzed *Gad2* mRNA transcript levels in *Drd2^Pet1^*^-CKO^ cells (dual *Drd2*-E7/8+ and *cre+* cells) in the DR (Fig. 7*C*). In males, we observed 87.44 ± 3.034% of *Drd2^Pet1^*^-CKO^ cells were *Gad2*+, while this percentage was 75.76 ± 0.5862% in females (*p* = 0.0157, unpaired *t* test; Fig. 7*D*). In these *Drd2^Pet1^*^-CKO^ cells, there were 14.25 ± 1.325 transcripts per cell in males and 10.13 ± 2.074 transcripts per cell in females (*p* = 0.1151, unpaired *t* test; Fig. 7*E*). Because of the tightly packed distribution of cells in the DR, puncta were measured only within *cre+* DAPI-stained nuclei to ensure puncta were not assigned to more than one cell. The area of nuclei did not differ between males (114.9 ± 3.030 μm^2^) and females (110.9 ± 1.768 μm^2^, *p* = 0.3497, unpaired *t* test; Fig. 7*F*). Thus, in *Drd2^Pet1^*^-CKO^ males as compared with *Drd2^Pet1^*^-CKO^ females, a greater percentage of the *Drd2-Pet1* cells harbored *Gad2* transcripts; of these *Gad2*-expressing cells, however, transcript levels were not significantly different between males versus female *Drd2^Pet1^*^-CKO^ mice.

**Figure 8.**
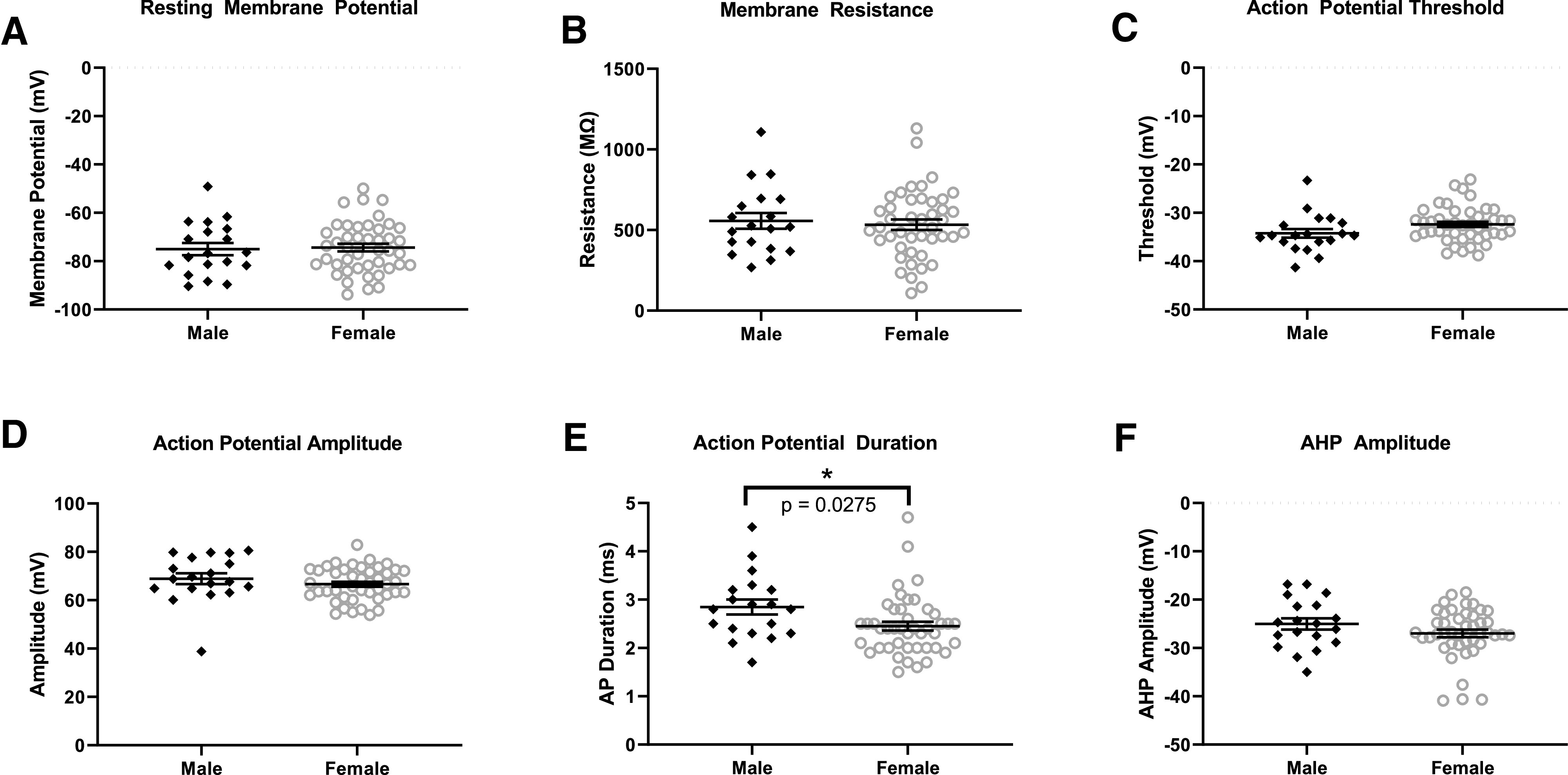
*Drd2-Pet1* neuron electrophysiological properties in male versus female mice. Membrane and AP characteristics were analyzed in GFP-marked *Drd2-Pet1* male and female neurons using whole-cell patch-clamp electrophysiology in acute brain slices from triple transgenic *Drd2-Cre;Pet1-Flpe;RC-FrePe* mice. Membrane potential (***A***), membrane resistance (***B***), AP threshold (***C***), AP amplitude (***D***), and AHP amplitude (***F***) do not differ in male (*n* = 19) or female (*n* = 44) *Drd2-Pet1* neurons while (***E***) male *Drd2-Pet1* neurons had a significantly longer (2.847 ± 0.155 ms, *n* = 19 cells) AP duration than in females (2.54 ± 0.094 ms, *n* = 44 cells, *p* = 0.0275, unpaired *t* test).

To explore potential sex differences in electrophysiological properties characterizing *Drd2-Pet1* neurons, we conducted whole-cell recordings from GFP-labeled *Drd2-Pet1* neurons in brain slices from triple transgenic *Drd2-Cre;Pet1-Flpe;RC-FrePe* males and females. Examination of cell membrane characteristics revealed no sex differences in resting membrane potential (AP; Fig. 8*A*) or resistance (Fig. 8*B*). Analyses of AP characteristics revealed an increase in AP duration (Fig. 8*E*) in male *Drd2-Pet1* cells as compared with female (2.847 ± 0.155 ms, *n* = 19 cells vs 2.54 ± 0.094 ms, *n* = 44 cells, respectively, *p* = 0.0275, unpaired *t* test], but no differences in AP threshold (Fig. 8*C*), amplitude (Fig. 8*D*), or afterhyperpolarization (AHP) amplitude (Fig. 8*F*).

### Differing covariance in axonal collateral densities from *Drd2-Pet1* neurons directed to auditory targets in males versus females

**Figure 9.**
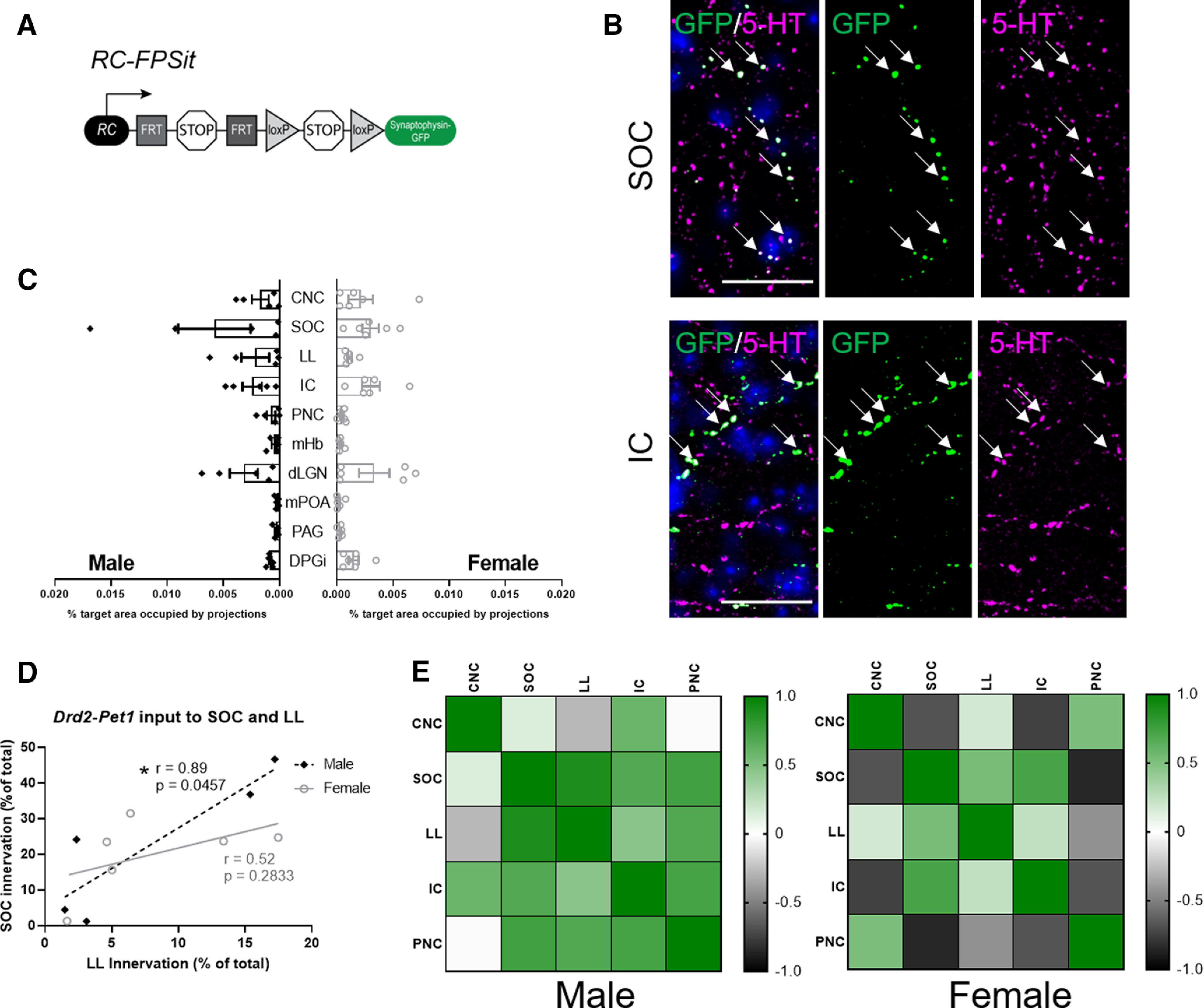
*Drd2-Pet1* neuron axon terminals target brain regions involved in sensory processing and defensive behavior in both male and female mice. ***A***, Intersectional genetic strategy: expression of *Drd2-Cre* and *Pet1-Flpe* transgenes results in dual recombination of intersectional allele, *RC-FPSit,* to label boutons of *Drd2-Pet1* neurons with Synaptophysin-GFP. ***B***, Representative images of *Drd2-Pet1* boutons in the SOC and IC. GFP+ (green, marked with arrows) boutons co-localize with 5-HT (magenta) staining. DAPI-stained nuclei shown in blue. Scale bar: 25 μm. ***C***, Quantification of the percent target area occupied by projections for all ten brain regions examined (for quantification protocol, see Materials and Methods). Target areas analyzed include brain regions involved in auditory processing and social behavior including the CNC, SOC, LL, IC, PNC, mHb, dLGN, mPOA, and PAG. The DPGi was also examined. No significant differences in projection area innervation were observed between males (*n* = 5) and females (*n* = 6). ***D***, Example graph showing correlation between innervation density of auditory brain regions differs in males compared with females. Each dot represents one animal. Values are shown as Pearson’s correlation coefficient (*r*), and * indicates *p* < 0.5 in a two-tailed test. ***E***, Pairwise correlations shown for male and female innervation density in auditory brain regions. Heatmaps represent high correlation (green) and low correlation (black) between CNC, SOC, LL, IC, and PNC.

As a first step in exploring sex differences in *Drd2-Pet1* neuron circuitry that may underlie the sex-specific behavioral phenotypes in *Drd2^Pet1-CKO^* mice, we compared relative innervation density to brain regions involved in sensory processing and social behavior in male and female mice. Boutons from *Drd2-Pet1* neurons were selectively marked with a Synaptophysin-GFP fusion protein using triple transgenic *Drd2-Cre;Pet1-Flpe;RC-FPsit* mice (Fig. 9*A*,*B*; Niederkofler et al., 2016). At P90, the same age at which the behavioral assays were conducted, we collected brain tissue and quantified projections to the cochlear nucleus complex (CNC), superior olivary complex (SOC), lateral lemniscus (LL), inferior colliculus (IC), caudal pontine reticular nucleus (PNC; critical for ASR; Davis et al., 1982), dorsal lateral geniculate nucleus (dLGN), mPOA, medial habenula (mHb), periaqueductal gray (PAG), and dorsal paragigantocellular nucleus (DPGi; shown as percentage of target area occupied by projections; Fig. 9*C*). We observed no significant sex differences in the cohort average for absolute innervation density to each of these 10 brain regions. However, because we observed considerable interanimal variability in bouton densities at targets, we next explored correlation of innervation density across brain regions (Weissbourd et al., 2014). Using pairwise correlations between auditory brain regions (Fig. 9*D*), we constructed a correlation matrix that shows positively correlated regions in green and negatively correlated regions in black (Fig. 9*E*). This visualization reveals that most auditory brain regions are positively correlated in males (SOC and LL, Pearson’s *r* = 0.89) with only the LL and cochlear nucleus being slightly negatively correlated (Pearson’s *r* = –0.28). Interestingly, a greater number of innervated regions were negatively correlated in females, including the CNC with both the SOC and the IC (*r* = −0.68 and *r* = −0.75, respectively), as well as PNC and IC (*r* = −0.67). The innervation of the PNC and SOC was significantly negatively correlated (*r* = −0.85, *p* = 0.033, two-tailed test). Further, we expanded analyses to include the dLGN, a region critical for visually-cued potentiation of the acoustic startle (Tischler and Davis, 1983), and found that in females innervation of the dLGN was not strongly correlated with innervation of auditory brain regions, while in males this dLGN innervation was highly negatively correlated with both the SOC (*r* = −0.91, *p* = 0.033, two-tailed test) and the IC (*r* = −0.91, *p* = 0.034, two-tailed test), indicating that *Drd2-Pet1* neuron circuitry may be set up to modulate multisensory information differently in males compared with females.

### GABA and 5-HT in *Drd2-Pet1* neurons

**Figure 10.**
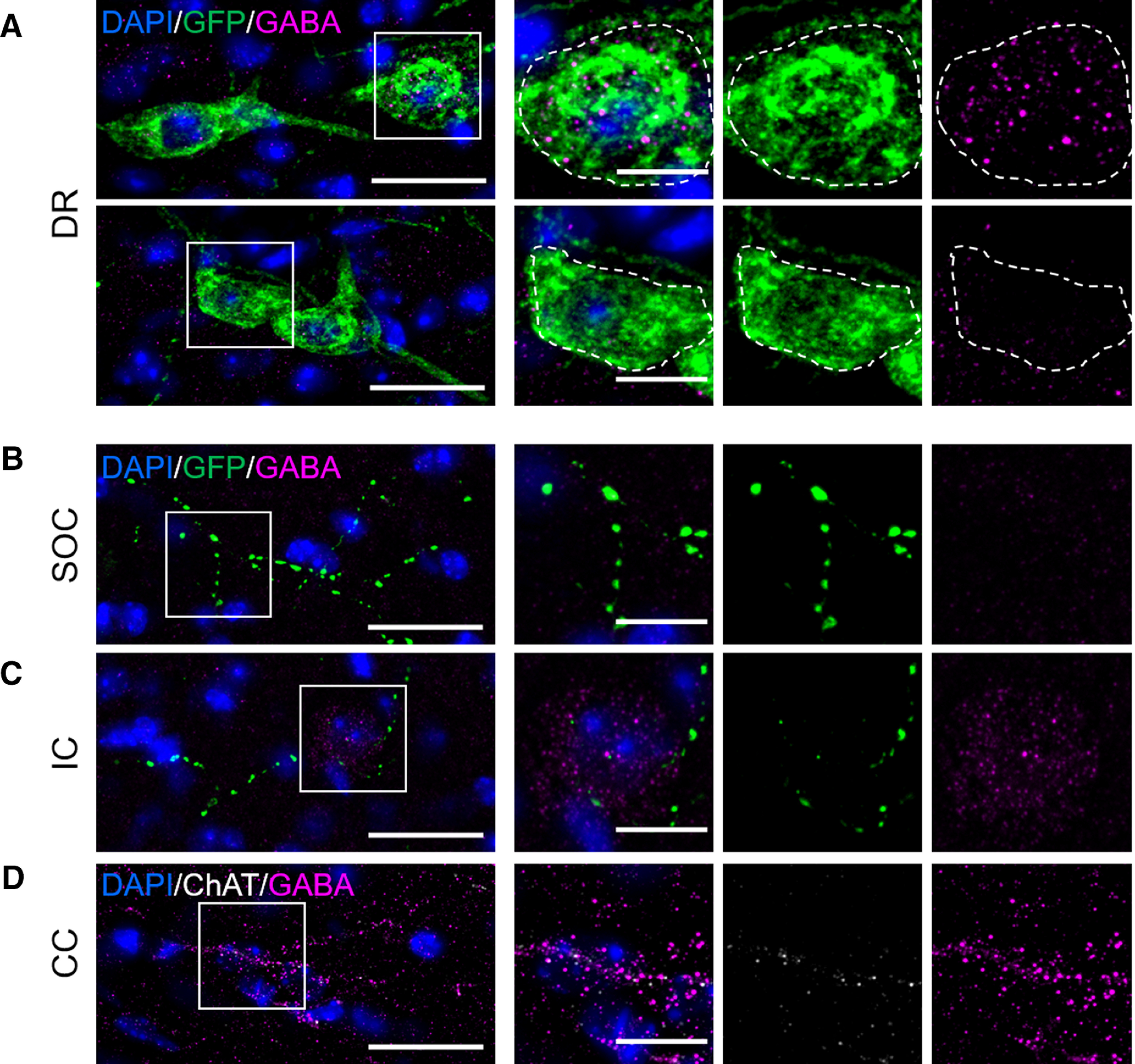
GABA immunoreactivity localizes to soma, but not axonal projections, of *Drd2-Pet1* neurons. ***A***, GABA staining (magenta) co-localizes with many *Drd2-Pet1* neuron soma (green GFP-positive cell bodies in *Drd2-Cre;Pet1-Flpe;RC-FPSit* mice) in the DR in a punctate manner (top), inset of boxed region showing neuron soma positive for GFP and GABA. Some *Drd2-Pet1* neuron soma are immuno-negative for GABA (bottom). Dotted lines encircle GFP-positive cell body. ***B***, ***C***, No GFP-positive *Drd2-Pet1* boutons (green) co-localize with GABA staining (magenta) in brain regions examined, shown here, representative images from SOC (***B***) and IC (***C***), noting a GABA-positive soma is visible in the image of the IC. ***D*,** GABA-positive immunoreactivity in the corpus callosum demonstrating detection of GABA boutons. ChAT (white) staining was used throughout for anatomic localization. Scale bars: 25 μm (left panel) and 10 μm (inset). DAPI-stained nuclei shown in blue.

Given detection of *Gad2* mRNA in *Drd2-Pet1* neurons, we probed for GABA versus 5-HT immunopositivity in cell soma versus axonal boutons in males versus females. Punctate GABA immunostaining was indeed detectable in some *Drd2-Pet1* neuron soma (Fig. 10*A*) in both males and females. Yet, in all target brain regions examined, GABA was undetectable in the GFP-marked *Drd2-Pet1* boutons. Shown are representative images from the SOC (Fig. 10*B*) and IC (Fig. 10*C*), noting a GABA-positive cell body in the IC (boxed) and GABA-positive staining in the corpus callosum serving as a positive control for GABA immunodetection (Fig. 10*D*). By contrast, 5-HT immunostaining in *Drd2-Pet1* boutons was readily detectable (representative images from the SOC and IC; Fig. 9*B*).

## Discussion

### Strategy

We hypothesized that loss of *Drd2* gene expression and associated DRD2 signaling normally observed in certain DR *Pet1^+^* serotonergic neurons (*Drd2-Pet1* neurons) could impair sensory, social, and/or defensive behaviors. We used the transgenic driver e*Pet-cre* to delete functionally critical *Drd2* gene sequences selectively in serotonergic neurons, thereby abolishing transcript and DRD2 protein function, which would normally initiate in *Pet1* cells during adolescence. We validated these *Drd2^Pet1-CKO^* mice and examined behavioral responses. Further, we explored *Drd2-Pet1* neurons themselves.

### Main findings

Key findings include the following. (1) Sex-specific behavioral alterations were observed in *Drd2^Pet1-CKO^* mice. Females showed a dramatic diminution in the protective, defensive ASR as compared with *Drd2^flox/flox^* controls, while no differences were observed in males. (2) *Drd2^Pet1-CKO^* males, but not females, showed increased winning in the tube test of social dominance against sex-matched and age-matched controls. (3) No differences were observed in ABRs, in PPI of acoustic startle, locomotion, cognition, nor various affective behaviors. (4) No sex-specific differences were found in *Drd2-Pet1* neuron number, soma distribution, nor in the set of efferent targets; however, within-animal correlations between efferent densities across target brain regions suggest differences by sex, thus hinting at sex-specific structural differences in *Drd2-Pet1* neuronal circuitry. (5) *Drd2-Pet1* cells in males as compared with females showed longer AP durations and higher levels of *Gad2* transcripts (important for GABA synthesis); *Drd2^Pet1^*^-CKO^ cells did not show a sex specific difference in *Gad2* transcript levels, but the percentage of *Drd2-Pet1* cells that were *Gad2^+^* in *Drd2^Pet1-CKO^* males was slightly higher than in *Drd2^Pet1-CKO^* females. These findings, coupled with our prior work (Niederkofler et al., 2016) implicating *Drd2-Pet1* neurons in setting levels of defensive aggressive and exploratory behaviors in male mice, suggest that *Drd2-Pet1* neurons may serve as a specialized neuromodulatory interface whereby DRD2 signaling alters serotonergic neuronal activity to shape defensive, protective, and dominance behaviors in a sex-specific manner.

### Protective ASR diminished in *Drd2^Pet1-CKO^* females

Defensive posturing in millisecond response to abrupt noise, be it a predator or other potential hazard, is a crucial evolutionarily conserved protective mechanism. Loss or blunting of this reflex can result in life-threatening exposure, while excessive enhancement can drive unnecessary, debilitating responses that preclude normal functioning. Thus, “tuning” of the ASR setpoint to social and environmental circumstances is likely critical for species survival and well-being. The observed ASR attenuation in female *Drd2^Pet1-CKO^* mice suggests that *Drd2-Pet1* neurons and the regulation of their activity cell autonomously by DRD2 comprises a critical modulatory node for ASR in females. Further, this node appears separate functionally from that involved in acoustic sensorimotor gating, given that acoustic PPI appeared intact in *Drd2^Pet1-CKO^* females, and from hearing, given that ABRs were indistinguishable from controls. Thus, DRD2 signaling in *Drd2-Pet1* neurons forms a functional circuit node specialized in female mice to influence startle to acoustic stimuli.

In rats, reduction of 5-HT through synthesis inhibition increases ASR in females, but not males (Pettersson et al., 2016). Predicted reciprocally is that elevated 5-HT levels might blunt ASR in females. Relating this to our findings, it is possible that *Drd2-Pet1* neurons are more excitable in the absence of DRD2-mediated inhibition, resulting in increased 5-HT release, perhaps explaining the observed ASR blunting. In wild-type mice, this would predict that under conditions of DA elevation, for example through local DR DA neuron activity associated with arousal and vigilance (Cho et al., 2017), *Drd2-Pet1* neuron activity would be inhibited, reducing 5-HT release and thereby tuning a more sensitive ASR, conferring a protective advantage.

The ASR circuit follows from cochlea to CNC to PNC to spinal motoneurons (Davis et al., 1982; Koch et al., 1992), and receives inputs from auditory centers such as the SOC, IC, and SC (Lauer et al., 2017). *Drd2-Pet1* neurons innervate each of these areas and the PNC, and thus may impart modulation at multiple levels.

### Tube test wins increased in *Drd2^Pet1-CKO^* males

The increased winning by *Drd2^Pet1-CKO^* males in the tube test suggests that loss of DRD2 results in an increase in or favoring of dominance behaviors, at least under these forced, one-on-one interaction conditions. We did not observe significant differences in levels of aggressive attack behaviors by *Drd2^Pet1-CKO^* males in a resident-intruder assay. Together, these findings suggest that in wild-type mice, DRD2 signaling in *Drd2-Pet1* neurons contributes to tempering certain dominance behaviors under particular conditions.

Understanding how the present results align with our prior work remains a pursuit. In earlier studies using a resident-intruder assay, we observed an increase in various aggressive behaviors in mice in which *Drd2-Pet1* neurons were constitutively silenced, which suggested to us that *Drd2-Pet1* neuron excitation and neurotransmitter release would normally temper such behaviors (i.e., favor non-confrontational, even submissive behaviors). Because canonical DRD2 signaling is inhibitory and, as well, appears largely inhibitory in *Drd2-Pet1* neurons in slice, we predicted that loss of DRD2 signaling would enhance *Drd2-Pet1* cell excitability and neurotransmitter release probability, and thus would suppress or at least not enhance dominance behaviors. Yet *Drd2^Pet1-CKO^* males exhibited enhanced winning in the tube test. Perhaps DRD2 signaling in *Drd2-Pet1* neurons results in cellular activity changes that ultimately lead to a tempering of one-on-one social dominance under some conditions, while extreme, constitutive *Drd2-Pet1* neuron silencing is required to prompt the opposite, in the form of aggression escalation to an intruder. Indeed, other findings also support this notion that dominance by tube test does not necessarily correlate with aggression in a resident-intruder assay (Tammimäki et al., 2010). Differences might also be explained by whether the input conditions trigger *Drd2-Pet1* neurons to release 5-HT versus GABA, should the latter prove a capability, noting that *Drd2-Pet1* cells express *Gad2*, albeit we were unable to show GABA in *Drd2-Pet1* boutons, only their soma.

Interestingly, a subset of *Drd2^Pet1-CKO^* males (four out of 26) did display increased levels of aggressive behaviors as compared with other *Drd2^Pet1-CKO^* mice and controls, suggesting there may be other influencing variables, yet unknown. This is plausible given that mice deficient for the long isoform of DRD2 (D_2L_R) are reported to show anxiety-like and depressive-like behaviors only following a stress-exposure paradigm (Shioda et al., 2019). Moreover, these stress-induced affective phenotypes in D_2L_R knock-out mice were abrogated by driving D_2L_R expression in DR *Pet1+* serotonergic neurons (Shioda et al., 2019). Together these findings suggest that the behavioral role of *Drd2* expression in *Drd2-Pet1* neurons may be influenced by environmental factors.

### Sex-specific differences in *Drd2-Pet1* neuron properties

The observed sex-specific differences in *Gad2* transcript levels in *Drd2-Pet1* neurons may contribute to the sex-specific behavioral alterations exhibited by *Drd2^Pet1-CKO^* mice. *Gad2* expression in *Drd2-Pet1* neurons is in line with prior reports showing *Gad2* expression more generally in the serotonergic DR (Nanopoulos et al., 1982; Calizo et al., 2011; Shikanai et al., 2012). It may be that *Drd2-Pet1* neurons can release GABA as well as or instead of 5-HT under certain conditions or at particular targets. This capacity may differ in males versus females, given our observation that in males, *Drd2-Pet1* neurons harbor higher levels of *Gad2* mRNA. Interestingly, *Drd2^Pet1^*^-CKO^ cells did not display this sex specific difference in *Gad2* transcript, suggesting that *Drd2* expression, or more broadly dopaminergic signaling in *Drd2-Pet1* neurons, may affect *Gad2* transcript levels. One potential model to be tested is if DRD2 signaling, in turn, alters levels of *Gad2* expression to allow for neuronal release of GABA in addition to or instead of serotonin when behavioral or environmental conditions necessitate. Indeed, there is precedent for the differential usage of serotonin and glutamate by raphe serotonergic neurons (Liu et al., 2014; Kapoor et al., 2016; Sengupta et al., 2017; Wang et al., 2019), although GABAergic and serotonergic co-release has not been reported.

AP duration measured *ex vivo* was longer in male versus female *Drd2-Pet1* cells; this may also confer neurotransmitter release properties that could contribute to behavioral differences. Additional studies are needed to determine how *Drd2^Pet1-CKO^* affects *Drd2-Pet1* neuron electrophysiology, gene expression, or efferent targets. Such experiments may be achieved through crossing *Drd2^Pet1^*^-CKO^ *(ePet1-Cre;Drd2^f/f^)* mice to *Drd2-Flpo* mice (The Jackson Laboratory strain #034419 provided by Bernardo Sabatini) along with an intersectional reporter transgene which would allow for dual Cre-mediated and Flp-mediated fluorescent labeling of mutant *Drd2^Pet1^*^-CKO^ cells. While complex genetics, this strategy would enable mutant cell visualization for electrophysiology, single-cell RNA sequencing, and analysis of axonal projections.

In both males and females, *Drd2-Pet1* neurons densely innervate auditory brainstem regions, likely modulating auditory-related processes at one or multiple of these sites. In examining *Drd2-Pet1* efferents, we observed interanimal variability in regional innervation density. We speculate this may arise from subgroups within the *Drd2-Pet1* neuron population that target different downstream structures. For example, some *Drd2-Pet1* neurons may project specifically to the SOC while others might project specifically to the LL. If some animals have more of one subgroup than the other, averaging absolute innervation densities for each target region across all males and females may hide meaningful circuit structure. Covariance analysis of projection targets in each animal thus might hint at which brain regions come under shared regulation by *Drd2-Pet1* neurons. In males, the high correlation between auditory region efferent densities suggests shared input from the same *Drd2-Pet1* neurons. In females, the CNC/SOC, CNC/IC, SOC/PNC, LL/PNC, and IC/PNC combinations were more negatively correlated, suggesting there might exist a subgroup of *Drd2-Pet1* neurons that targets the PNC and a different subgroup, the SOC. We speculate that in males, *Drd2-Pet1* neurons contribute to a general level of serotonergic tone across the auditory brainstem, while in females, certain *Drd2-Pet1* neurons selectively target and modulate specific nuclei.

In conclusion, we found that *Drd2* gene expression in a specialized subset of *Pet1* serotonergic neurons is required for certain defensive, dominance, and protective behaviors, involving auditory processing in a sex-specific manner. Deficits in sensory processing such as altered acoustic startle and impaired social communication and dominance behaviors manifest in human disorders including autism spectrum disorder, schizophrenia, and posttraumatic stress disorder, often in sex-specific ways (King et al., 2013; Steel et al., 2014; Matsuo et al., 2016; Thye et al., 2018) and with sex-specific differences in therapeutic outcomes (Franconi et al., 2007). The presented findings, thus, may point to novel circuit nodes of relevance to human neuropsychiatric disease.

## Synthesis

Reviewing Editor: Christie Fowler, University of California, Irvine Decisions are customarily a result of the Reviewing Editor and the peer reviewers coming together and discussing their recommendations until a consensus is reached. When revisions are invited, a fact-based synthesis statement explaining their decision and outlining what is needed to prepare a revision will be listed below. The following reviewer(s) agreed to reveal their identity: David Dietz, Mary (Molly) Heyer. Note: If this manuscript was transferred from JNeurosci and a decision was made to accept the manuscript without peer review, a brief statement to this effect will instead be what is listed below.

The authors have done an excellent job addressing the reviewers’ comments, and they should be commended for their detailed attention to statistical and imaging analyses. The discussion section has also been improved by their thoughtful additions.

## Author Response

Re: Resubmission of manuscript eN-TNWR-0202-20X: Sex-specific role for dopamine receptor D2 in dorsal raphe serotonergic neuron modulation of defensive acoustic startle and dominance behavior.

We have uploaded a revised version of our manuscript eN-TNWR-0202-20x. The reviews were insightful and thoughtful; our addressing of each point has indeed raised substantially the manuscript’s scholarly level. We appreciate the opportunity to submit this revision, including the below detailed explanation of our responses.

Comments are addressed in the order by which they appeared in the eNeuro decision letter.

1. While the significance statement contained valid statements, it is not written grammatically correct and thus needs revision

Response: Thank you, the significance statement has been rewritten.

2. Synthesis Statement: The main concern pertains to the indication that tools are not available to examine sex-specific differences following Drd2 deletion in Drd2-Pet1 cells. However, a custom RNAscope probe specific to the mRNA with the deleted exon could be used for in situ colocalization studies - this could be done in a reasonable time frame with minimal additional animal usage. While other approaches (e.g., single cell sequencing, alternate breeding strategies) were discussed by the reviewers as having the potential to provide more enriched data sets, they recognized that these approaches would require much time/effort and could just be highlighted in the discussion as a potential future experiment.

Response: We appreciate the ideas and way of thinking embodied in the Synthesis Statement. Accordingly, we have followed up with additions - experimentally and in the text - to our revision that are in line with these points. Greater detail follows, point-by-point below.

3. As currently written, this manuscript appears to lack a mechanistic central hypothesis. This is not to say the data are not worthy of publication or that there is an inherent issue, but currently the overall importance of these findings is lost in what seems like a phenotyping endeavor. Indeed, I suspect that based on similar publications in 2016 that the authors had more of concept of why these findings fit well.

Response: Revisions now highlight the hypothesis more clearly; we thank the reviewers for their careful attention and suggestion.

4. The only methodical concern appears to be the use of genetically identical mice in the tube test. The appropriate measure would have been to utilize a control non- genetic mutant as one of the test subjects. Having said that, the data from this pairing is interesting and should not be excluded.

Response: Within the literature, there are multiple methods reported for conducting the tube test. We conducted ours in accordance with the Harvard NeuroBehavior Core’s instructions and similar to other papers that use wild-type littermate controls against a mutant mouse (in our case, a control mouse harboring the floxed Drd2 allele but not expressing Cre paired against the experimental Drd2Pet1-CKO opponent - noting that Drd2flox/flox mice do not have altered social dominance1). References 2 and 3 below use similar tube test methodology as employed here.

1. Daniela Noaín, M. Inés Pérez-Millán, Estefanía P. Bello, Guillermina M. Luque, Rodrigo Casas Cordero, Diego M. Gelman, Marcela Peper, Isabel García Tornadu, Malcolm J. Low, Damasia Becú-Villalobos, Marcelo Rubinstein. (2013). Central Dopamine D2 Receptors Regulate Growth-Hormone-Dependent Body Growth and Pheromone Signaling to Conspecific Males. Journal of Neuroscience, 33 (13) 5834-5842; DOI: 10.1523/JNEUROSCI.5673-12.2013

2. Zhou, Y., Kaiser, T., Monteiro, P., Zhang, X., Van der Goes, M. S., Wang, D., Barak, B., Zeng, M., Li, C., Lu, C., Wells, M., Amaya, A., Nguyen, S., Lewis, M., Sanjana, N., Zhou, Y., Zhang, M., Zhang, F., Fu, Z., & Feng, G. (2016). Mice with Shank3 Mutations Associated with ASD and Schizophrenia Display Both Shared and Distinct Defects. Neuron, 89(1), 147-162. https://doi.org/10.1016/j.neuron.2015.11.023

3. Wei-Hsiang Huang, David C. Wang, William E. Allen, Matthew Klope, Hailan Hu, Mehrdad Shamloo, Liqun Luo. (2018). Early adolescent Rai1 reactivation reverses transcriptional and social interaction deficits in a mouse model of Smith-Magenis syndrome. PNAS, 115 (42) 10744-10749; DOI: 10.1073/pnas.1806796115

5. The n for some of the molecular endpoints is very low (i.e. 3)

Response: The N for RNAscope studies in wildtype tissue has been increased from 3 to 5.

6. Given the very large n for some of the behavioral experiments--did the authors note any influence on cycle in female mice as has been reported previously?

Response: We did not examine estrous cycle in females given that published findings using C57BL/6J (the genetic background of mice used in our studies) indicate: no effect of estrous cycle in C57BL/6J on the acoustic startle reflex (Meziane et al., 2007); no effect on open field, rotarod, and pre-pulse inhibition of acoustic startle, noting that tail suspension test did show increased immobility in the metestrus phase (Meziane et al., 2007); see also Palanza et al., 2001; Romeo et al., 2003 found no effect of estrous cycle in open field; Plappert et al., 2005 found no effect of estrous cycle for acoustic startle response and prepulse inhibition.

7. Please include the Dorsal Raphe in the title.

Response: Thank you, the title has been changed to ‘Sex-specific role for dopamine receptor D2 in dorsal raphe serotonergic neuron modulation of defensive acoustic startle and dominance behavior’

8. The main weakness of the paper is that the role of Drd2 in serotonergic neurons is only explored in the context of sex-specific behavioral differences following conditional Drd2 deletion. Gene expression, electrophysiology, and connectivity data from this population of neurons are only studied in wild type mice, such that any findings cannot be directly linked to the sex-specific effects of Drd2 deletion. There are a wide range of sex-specific behavioral differences in WT mice, so the usefulness of these data as pertains to the behaviors in question is unclear

Response: Please see below (point #9).

9. The authors state that tools are not available to examine sex-specific differences following Drd2 deletion in Drd2-Pet1 cells, as the ePet-Cre driver used for deletion of Drd2 would activate reporter/effector/indicator alleles in all Pet1+ neurons. However, single cell sequencing could be performed after Drd2 deletion in this cell type, which would be demarcated by the mutated Drd2 mRNA missing the floxed exon, as long as the mRNA is not degraded/destabilized. Similarly, a custom RNAscope probe specific to the mRNA with deleted exon could be used for in situ colocalization studies. In addition, if the authors were to swap their original genetic strategy for reporter/effector expression in Drd2-Pet1+ neurons by using the Drd2-Flp line and Pet1-Cre line, reporter expression AND Drd2 deletion could be achieved in the same mice, thus allowing for more thorough analysis of sex-dependent differences in this population that could be linked directly to the sex differences in behavior.

Response: We thank the reviewers for their insights into probing gene expression in Drd2Pet1-CKO cells. In line with the synthesis statement, we pursued the use of a combination of RNAscope probes to identify Drd2Pet1-CKO (knockout) cells. Cre-mediated deletion removes exon 2 from the Drd2 gene and subsequent transcript, leaving more distal exons, such as 7 and 8, intact. Assuming little degradation of the generated mutant Drd2 transcript, we tested the strategy that CKO cells would be identifiable as hybridization-positive for probes for Drd2-Exon7/8 and for cre mRNA but not Drd2-Exon2; by contrast, wild-type cells would be positive for Drd2-Exon7/8 and Drd2-Exon2 probes. After rigorous validation of a Drd2-Exon7/8 RNAscope probe in both control and knockout tissue, we paired this probe with a cre-specific probe to identify ‘CKO cells’ in Pet1Drd2-CKO mice. We then analyzed CKO cells for altered levels of Gad2 mRNA, given our finding of increased expression in wild-type male versus female Drd2-Pet1 neurons.

The Reviewer suggestions involving single-cell RNA sequencing and alternative breeding strategies are valuable, creative, and elegant, have not eluded us, and are discussed in this revision as future directions, in line with the editor recommendation found in the Synthesis statement. These experiments involve extensive time, labor, and cost, putting them outside the scope of this submission. Entailed would be additional transgenes and entirely new breeding strategies. For example, single-cell RNA sequencing experiments would likely require the addition of another transgene (a Cre-dependent fluorescent reporter) to the Drd2Pet1-CKO genotype to allow for selecting serotonergic neurons, which otherwise would be poorly represented among brainstem cells given that they are few in number (see recent yields by Huang et al., eLife 2019). Moreover, within such a pool of manually- or On-Chip-sorted fluorescently marked serotonergic neurons (Okaty et al., 2020 eLife), those of the Drd2-Pet1 subtype are even fewer (∼400-500 neurons per animal). As well, deep sequencing is required to detect Drd2 transcripts, let alone mutant Drd2 transcripts needed as the key cell identifier.

Regarding the reverse recombinase strategy involving the Drd2-Flpo transgenic, we have indeed been working with that line, validating its specificity and thus appropriateness for the suggested experiments. Jackson Laboratories describes this line as, “Specificity is described as ‘imperfect’ by the donating laboratory.’ We look forward to continuing along this avenue of investigation, but at present, feasibility remains to be demonstrated.

10. The figure legend for Figure 5 has incorrect letters for the corresponding panel (E should be D, etc). Also, in panel E, the data points at the 100% mark appear to be cropped.

Response: Thank you, this figure has been updated.
